# Rapid response to anthropogenic climate change by *Thuja occidentalis*: implications for past climate reconstructions and future climate predictions

**DOI:** 10.7717/peerj.7378

**Published:** 2019-07-26

**Authors:** Rebekah A. Stein, Nathan D. Sheldon, Selena Smith

**Affiliations:** Department of Earth and Environmental Sciences, University of Michigan–Ann Arbor, Ann Arbor, MI, USA

**Keywords:** Atmosphere, Biogeochemistry, Carbon isotopes, Northern white cedar, Biosphere, Terrestrial, Climate, Paleoclimate

## Abstract

Carbon isotope values of leaves (δ^13^C_leaf_) from meta-analyses and growth chamber studies of C_3_ plants have been used to propose generalized relationships between δ^13^C_leaf_ and climate variables such as mean annual precipitation (MAP), atmospheric concentration of carbon dioxide ([CO_2_]), and other climate variables. These generalized relationships are frequently applied to the fossil record to create paleoclimate reconstructions. Although plant evolution influences biochemistry and response to environmental stress, few studies have assessed species-specific carbon assimilation as it relates to climate outside of a laboratory. We measured δ^13^C_leaf_ values and C:N ratios of a wide-ranging evergreen conifer with a long fossil record, *Thuja occidentalis* (Cupressaceae) collected 1804–2017, in order to maximize potential paleo-applications of our focal species. This high-resolution record represents a natural experiment from pre-Industrial to Industrial times, which spans a range of geologically meaningful [CO_2_] and δ^13^C_atm_ values. Δ_leaf_ values (carbon isotope discrimination between δ^13^C_atm_ and δ^13^C_leaf_) remain constant across climate conditions, indicating limited response to environmental stress. Only δ^13^C_leaf_ and δ^13^C_atm_ values showed a strong relationship (linear), thus, δ^13^C_leaf_ is an excellent record of carbon isotopic changes in the atmosphere during Industrialization. In contrast with previous free-air concentration enrichment experiments, no relationship was found between C:N ratios and increasing [CO_2_]. Simultaneously static C:N ratios and Δ_leaf_ in light of increasing CO_2_ highlights plants’ inability to match rapid climate change with increased carbon assimilation as previously expected; Δ_leaf_ values are not reliable tools to reconstruct MAP and [CO_2_], and δ^13^C_leaf_ values only decrease with [CO_2_] in line with atmospheric carbon isotope changes.

## Introduction

The concentration ([CO_2_]) and isotopic value (δ^13^C_atm_) of atmospheric CO_2_ are changing at a pace unprecedented in geologic time (Keeling et al., 2005; Zhang et al., 2013). These changes have been accompanied by regional changes in mean annual temperature (MAT), mean annual precipitation (MAP), maximum summer temperature, and other climate variables (Yonetani & Gordon, 2001). The rapid decline in the carbon isotopic composition of CO_2_ (δ^13^C_atm_) due to fossil fuel combustion, deforestation, and other human inputs, is known as the Suess Effect, and is a chemical representation of anthropogenic changes to the atmosphere—and more broadly, the environment. δ^13^C_atm_ values provide a useful way to see changes in CO_2_ sources, sinks, and fluxes in the modern environment (Keeling, 1979; Boutton, 1991; Deines, 1992). It can also be applied to geologic problems (Schmitt et al., 2012) due to the naturally differing isotopic compositions of different CO_2_ sources (e.g., methane, volcanism). δ^13^C_atm_ values are particularly useful because they are parameters in models that reconstruct past changes to atmospheric [CO_2_] using paleosol carbonates (Cerling et al., 1991, 1992) or atmospheric [CO_2_] using plant stomatal parameters (Franks et al., 2014). Direct measurements of δ^13^C_atm_ values only go back 50 years due to technological limitations, and longer-reaching ice core CO_2_ bubbles (∼800,000 years) are poorly resolved for recent times and limited by the presence of glacial ice (Keeling & Whorf, 2004; Augustin et al., 2004; Barnola et al., 1987; Trudinger et al., 1999; Petit et al., 1999). The biosphere provides an excellent system that directly interacts with the atmosphere and fills the gap to provide high-resolution recent and long-term records, potentially extending into geologic time (Arens, Jahren & Amundson, 2000).

This direct interaction means that plants potentially provide a robust record of δ^13^C_atm_ values in their own leaf carbon isotope values (δ^13^C_leaf_) and fractionation values (Δ_leaf_, Eq. (1); Farquhar, Ehleringer & Hubick, 1989; Feng, 1999; Farquhar & Sharkey, 1982), which gives insight into changes in carbon assimilation over time. In Eq. (1), *a* represents the fractionation of δ^13^C due to diffusion in air (4.4‰) and *b* represents the fractionation due to the carboxylation (instigated by the Rubisco enzyme, 27‰; Farquhar, Ehleringer & Hubick, 1989). These fractionation factors are compiled and multiplied by the ratio of *C_i_* (intercellular [CO_2_]) to *C_a_* (atmospheric [CO_2_]), a ratio that is often used to represent water use efficiency.

(1) $${\Delta _{{\rm{leaf}}}} = {{({{\rm{\delta }}^{13}}{{\rm{C}}_{{\rm{atm}}}} - {{\rm{\delta }}^{13}}{{\rm{C}}_{{\rm{leaf}}}})} \over {\left( {1 + {{\rm{\delta }}^{13}}{{\rm{C}}_{{\rm{leaf}}}}/1000} \right)}} = a + \left( {b - a} \right)\left( {{{{C_i}} \over {{C_a}}}} \right)$$

While *a* and *b* are thought to be constant, we know that δ^13^C_atm_ and *C_a_* are changing rapidly. This could result in a corresponding change in Δ_leaf_ values as plants adapt to increased [CO_2_] or subsequent regional climate changes, for example, systematic changes in local precipitation). Alternatively, Δ_leaf_ values of leaves may stay constant but show marked changes in δ^13^C_leaf_ values corresponding to changes in δ^13^C_atm_ values if leaves are incorporating δ^13^C_atm_ into leaf tissues at a rate unaffected by other climate conditions.

## Carbon Isotopes Related to Climate Variables

Previous studies have related Δ_leaf_ values to climate variables such as MAP, water availability and soil moisture (Diefendorf et al., 2010; Kohn, 2010; Wernerehl & Givnish, 2015; Mårtensson et al., 2017), MAT (Troughton & Card, 1975; O’Leary, 1993); latitude (Diefendorf et al., 2010; Kohn, 2010), [CO_2_] (Schubert & Jahren, 2012, 2018), altitude (Korner, Farquhar & Wong, 1991; O’Leary, 1993), seasonality (Ehleringer, Phillips & Comstock, 1992), and δ^13^C_atm_ values during growth seasons (Peñuelas & Azcón-Bieto, 1992; Arens, Jahren & Amundson, 2000; Pedicino et al., 2002). The studies that incorporate potential influence from a wide range of climate variables have been conducted via meta-analysis with no normalized collection procedure or investigated species, or via growth chamber experiment conducted under idealized conditions. The few studies that have used naturally-obtained specimens (i.e., natural history collections such as herbaria) to look at isotope change over time and changing atmospheric conditions ([CO_2_] and δ^13^C_atm_ values) have focused on localized regions with little range in climate (i.e., all dry, mid- to high- altitude, hot regions of eastern Arizona/western New Mexico, or the Mediterranean climate of Catalonia; Peñuelas & Azcón-Bieto, 1992; Pedicino et al., 2002). These collections-based experiments are limited in scope and while they provide information on specific ecosystems, do not address these biosphere-atmosphere interactions across climate regimes or on a regional and global scale. Very little is known about whether individual species respond to any, some, or all of these potential forcings across a range of climatic conditions.

## Potential Climate Drivers

### [CO_2_] and elevation

We would expect higher [CO_2_] to affect biochemical discrimination because of the known effects elevated [CO_2_] has on stomata (size, density, and conductance; Woodward, 1987; Woodward & Bazzaz, 1988; Tognetti et al., 2000; Ainsworth & Rogers, 2007). In a meta-analysis of trees in European temperate and boreal forests, leaves responded to an increase in [CO_2_] with a significant (21%) decrease in stomatal conductance (the rate of passage of atmospheric CO_2_ into plant tissue; Medlyn et al., 2001). Increased [CO_2_] also causes a decrease in stomatal density (the number of pores on a leaf surface) and stomatal index (the number of pores compared to the number of total epidermal cells); these indices have been liberally applied to the geologic record to reconstruct [CO_2_] (Retallack, 2001; Beerling & Royer, 2002; Roth-Nebelsick et al., 2014). Because stomata directly control the flow of carbon dioxide into leaves and control carbon isotope fractionation by diffusion (Farquhar, Ehleringer & Hubick, 1989), changes in stomatal parameters could affect fractionation as well. Additional factors must be considered; the dependence of Δ_leaf_ on [CO_2_] may in part be due to isotopic discrimination associated with photorespiration (Schubert & Jahren, 2018). Indeed, previous growth chamber studies in prescribed CO_2_ environments showed increased carbon isotope fractionation with increased [CO_2_]; Schubert & Jahren (2012) found a strong hyperbolic correlation (*r* > 0.94) between [CO_2_] and Δ_leaf_ values in two species of herbaceous angiosperms. Based upon these growth chamber experiments (Schubert & Jahren, 2012, 2018), the relationship between [CO_2_] and Δ_leaf_ is expected to be most sensitive at geologically low [CO_2_] (including pre-Industrial to present values) as it was in the levels present during plant growth in this study.

Elevation has been shown to factor into carbon isotope discrimination but is frequently not evaluated independently, due to its covariant relationship with climate variables such as temperature, vapor pressure, partial pressure of CO_2_ (*p*CO_2_), soil [CO_2_], and soil texture (Diefendorf et al., 2010). A study looking at *Salix herbacea* leaves along an altitudinal gradient (2,000–2,800 m) in Austria showed a decrease in carbon isotope value with increased altitude, did not account for corresponding changes in other climate variables (Beerling, Mattey & Chaloner, 1993). Another study done in Utah and New Mexico using a number of desert and woodland species, including angiosperms and gymnosperms, found similar negative trends in δ^13^C_leaf_ with increased altitude without controlling for other climate variables (Van de Water, Leavitt & Betancourt, 2002). In 2010, a meta-analysis assessing carbon isotope fractionation and discrimination values across a wide range of C_3_ plants (Diefendorf et al., 2010) found that when combined with MAP, elevation explained 61% of variability in Δ_leaf_ values. Based upon these studies, we included elevation as a potential variable in our study, however, we chose sample locations to minimize changes in elevation because it is difficult to completely separate this variable from regional variation in [CO_2_] and δ^13^C_atm_.

### δ^13^C_atm_

With the current increase in [CO_2_], we have observed the aforementioned Suess Effect, wherein δ^13^C_atm_ has changed in response to increased inputs of more isotopically negative CO_2_ into the atmosphere (Keeling, 1979). The composition of CO_2_ involved in the making of organic tissue is likely to affect the composition of that organic tissue (Arens, Jahren & Amundson, 2000); our study will test if this effect is compounded or mitigated by changes in other climate variables over this chronologically robust natural experiment.

### Latitude

Latitude is expected to affect stomatal traits and therefore be related to carbon isotope fractionation due to its inverse relationship with light (specifically length of growing season and length of day) and temperature, and consequent effects on the maximum operating times for photosynthesis. A meta-analysis across 760 species in nine Chinese forest ecosystems showed a latitudinal variation in stomatal density and stomatal length at the community level (Wang et al., 2015). Given these morphological changes due to latitude, and the relationship between latitude and temperature, we might expect that Δ_leaf_ values would be inversely related to latitude as well (Farquhar, Ehleringer & Hubick, 1989; Eq. (1)). The relationship between Δ_leaf_ and latitude (15.9°S through 69.5°N) was observed in results of a meta-analysis of plants that used C_3_ photosynthetic pathways (*n* = 506) (Diefendorf et al., 2010), but any changes in Δ_leaf_ as a function of latitude disappeared when latitude was decoupled from temperature and precipitation.

### Precipitation

The stomata act as an inlet for CO_2_ uptake as well as an outlet for leaf water loss via transpiration, which is why one might expect a relationship between carbon isotope fractionation and available water (represented in the paleo-record as reconstructed MAP). With decreased available water (i.e., decreased MAP) comes increased need for plant “water use efficiency” (as measured by the ratio of water used in photosynthesis to water lost through transpiration); plants therefore minimize water loss through the same stomata by fully closing, resulting in decreased carbon isotope fractionation (Farquhar, Ehleringer & Hubick, 1989). Previous meta-analyses, such as those by Diefendorf et al. (2010) and Kohn (2010), compared Δ_leaf_ values of a wide variety of modern C_3_ plants from many regions with MAP and found that Δ_leaf_ varied significantly with MAP (*p*-value = 0.0001 and *R*^2^ = 0.57; Diefendorf et al., 2010).

### Temperature and seasonality

In addition to the climate variables with pre-established and applied relationships with Δ_leaf_ values ([CO_2_], MAP), various hypotheses have been proposed about the relationships between carbon isotope discrimination and other climate variables. For example, MAT could also constrain photosynthetic processes and associated carbon isotope fractionation because it gives a rough representation of extreme conditions and growing season length during which carbon assimilation occurs. For this reason, MAT is a well-addressed climate variable in previous isotope fractionation studies, but none have identified a relationship between fractionation and MAT (Arens, Jahren & Amundson, 2000; Diefendorf et al., 2010; Kohn, 2010; Schubert & Jahren, 2012). Furthermore, Helliker & Richter (2008) found that leaves maintained a constant internal temperature ideal for photosynthesis of 21.4 ± 2.2 °C (total range of measurements), independent of external temperatures. In addition to MAT, maximum summer temperatures (particularly, the extreme highs associated with a warming climate) are expected to increase (Mirza, 2003). Increased maximum summer temperatures lead to increased evapotranspiration and more plant stress, which might affect carbon assimilation rates and stomatal conductivity (Farquhar, Ehleringer & Hubick, 1989; Diefendorf et al., 2010).

Seasonal variation is also thought to affect isotopic discrimination, resulting in a change in δ^13^C_leaf_ of up to 1–2‰ (Ehleringer, Phillips & Comstock, 1992; Arens, Jahren & Amundson, 2000), or in some deciduous trees such as maples, up to 6‰ between early spring and late fall (Lowdon & Dyck, 1974). Typically, more positive values are found in the winter (indicative of less isotopic discrimination) and more negative values occur in the summer (indicative of more discrimination). This effect strongest in arid and semiarid environments because they experience amplified seasonal temperature, precipitation, and evaporation effects (up to 4‰; Ehleringer, Phillips & Comstock, 1992). Our choice of sample locations should minimize this effect, as our specimens come from humid regions.

Though we do not expect a correlation between temperature and seasonality with Δ_leaf_ values, nor are there good proxies for these variables in the fossil record, we include them here for completeness. This ensures that any observed noise is random or unaccountable for in the fossil record, rather than related to variables that are commonly measured and potentially relevant in plant isotope discrimination (Farquhar, Ehleringer & Hubick, 1989; Arens, Jahren & Amundson, 2000).

### Finding a focal species

In addition to potentially confounding climate variables, variation in δ^13^C_leaf_ and Δ_leaf_ values can be related to species-inherent carbon isotope fractionation. In a recent meta-analysis of C_3_ plants conducted by Diefendorf et al. (2010), Δ_leaf_ values ranged from 13.4‰ for *Pinus edulis* in Utah, USA (Van de Water, Leavitt & Betancourt, 2002) to 28.4‰ for *Cryptocarya concinna* in Guangdong Province, China (Ehleringer, Phillips & Comstock, 1992). However, the focus on geographic and climatic variability within that dataset resulted in a small number of analyses of any individual species. Thus, while some previous studies have proposed a universal Δ_leaf_ value that represents C_3_ plants on average (Arens, Jahren & Amundson, 2000; Gröcke, 2002), we focus here instead on an individual species (*Thuja occidentalis*; Cupressaceae) in order to avoid interspecific variation and phylogenetic/evolutionary effects in plant biochemistry.

*Thuja occidentalis* is a widespread evergreen gymnosperm with a distribution today extending throughout temperate deciduous and boreal forests in North America, and an extensive fossil record in localities across North America dating back to the Late Cretaceous (∼71 million years ago; LePage, 2003; Eckenwalder, 2009). *T. occidentalis* leaves have longer life spans (>1 year) than deciduous trees (Givnish, 2002; Pease, 1917), which makes them less vulnerable to seasonal variability and hardier in sedimentary archives (Diefendorf, Leslie & Wing, 2015). While some studies of other individual species have demonstrated unexplained internal isotopic variation of up to 3‰ (Tieszen, 1991), Mervenne (2015) found that Δ_leaf_ values of *T. occidentalis* showed the least amount of internal isotopic variation within a single species grown in a common garden site (e.g., 18.91 ± 0.46‰ vs. *Taxus:* 20.05 ± 1.93‰) when compared to 56 species native to temperate forests. This makes *T. occidentalis* an excellent focal taxon for a single-species study.

This study incorporates the natural shifts in [CO_2_] concentrations as driven by fossil fuel combustion and other anthropogenic inputs since the Industrial Revolution (280 ppm: Pre-Industrial, to ∼410 ppm in 2014; IPCC, 2014) and δ^13^C_atm_ values (from −6.5‰: Pre-Industrial to −8.5‰: present; Araus & Buxó, 1993; Elsig et al., 2009; White, Vaughn & Michel, 2015) to examine the relationship between *T. occidentalis*’ carbon isotope fractionation and leaf chemistry (C:N ratios) within a range of climate variables. The patterns of Δ_leaf_ values over a range of δ^13^C_atm_ values highlight the limitations of δ^13^C_leaf_ change as a tool for better understanding the biosphere and atmosphere. If δ^13^C_leaf_ values change in sync with δ^13^C_atm_ values, this could mean that carbon assimilation is continuing in this species as it was prior to Industrialization, perhaps indicating *T. occidentalis*’ lack of adaptation to increased [CO_2_]. Additionally, it may indicate that δ^13^C_leaf_ provides another way to track anthropogenic changes to the environment in the recent past and the future.

## Materials and Methods

**Figure 1:**
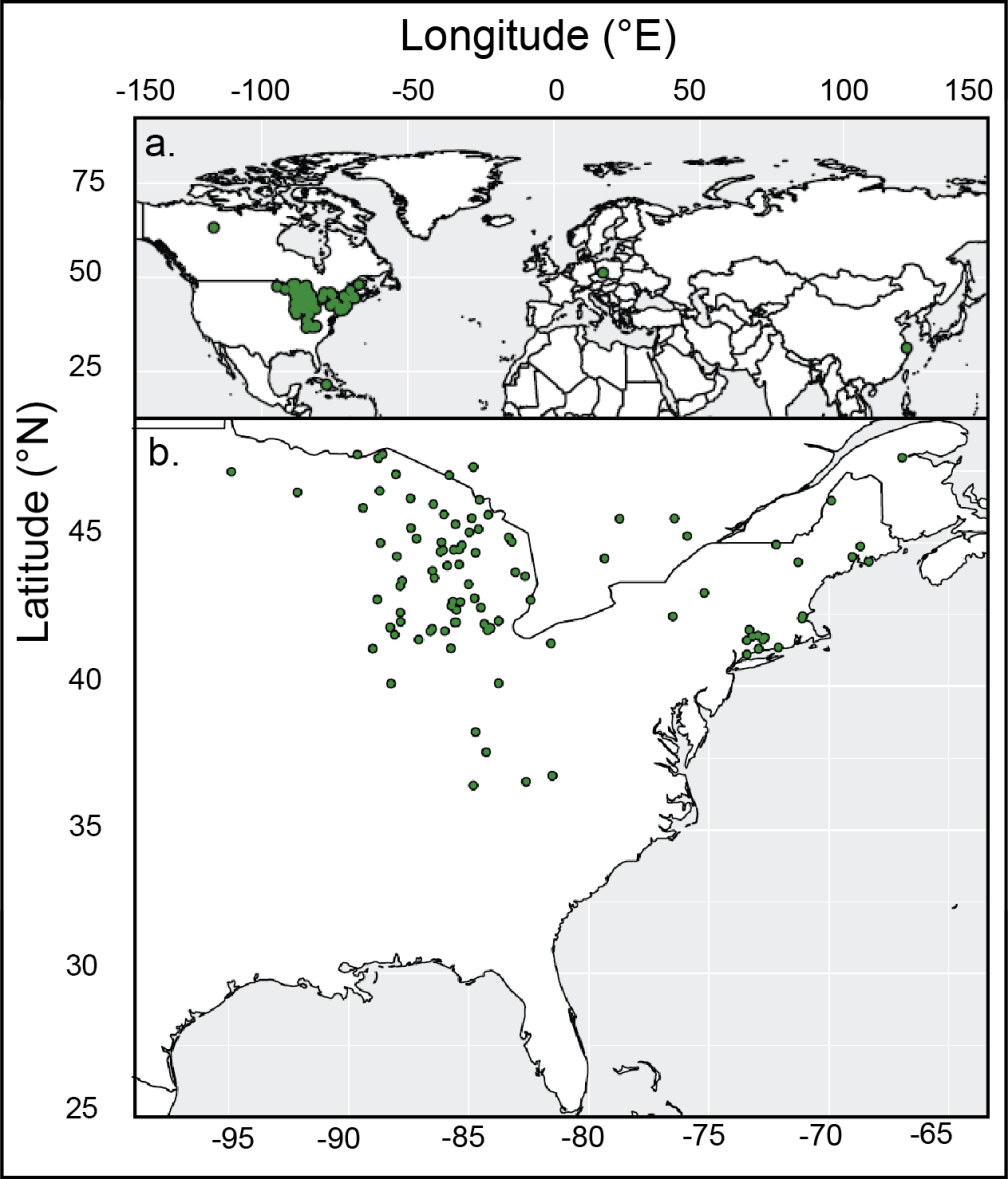
Map of locations of *Thuja occidentalis* specimens. Locations of (A) collections of *Thuja occidentalis* from across the world. (B) Specimens collected in eastern North America (in the typical habitat of *Thuja occidentalis*).

We measured δ^13^C_leaf_ values of *T. occidentalis* extending from present-day to Pre-Industrial historical records using both newly collected and herbarium material. This included collecting leaf material of *T. occidentalis* specimens (*n* = 142 collected between 1804 and 2017 with four unknown dates of collection) from across the Great Lakes region from herbaria (Figs. 1A and 1B; Table S1) and in natural present-day occurrences across a range of climate conditions (see Table S2). *Thuja* has small, 1–10 mm long scale-leaves adpressed along a small branch. Three cm portions of branches representing a single growth year with multiple scale-leaves were cleaned in an ultrasonic bath of deionized water to remove surface debris, oven dried at 50 °C for 48 h and homogenized; this removes any within-leaf isotopic variation. Aliquots of each *T. occidentalis* specimen (0.6–0.8 mg) were placed into tin capsules and placed in a Costech elemental analyzer to measure %C and %N (as well as C:N ratio). Second aliquots of each *T. occidentalis* specimen (0.6–0.8 mg) were placed into tin capsules and placed in a combustion module inlet coupled to a Picarro G2201-i cavity ring-down spectrometer (CRDS) to measure δ^13^C values of each specimen. Duplicates were run on both machines to insure homogeneity. Results of each CRDS run were internally calibrated using nine acetanilide standards (δ^13^C = −28.17 ± 0.16‰), two IAEA-600 caffeine standards (δ^13^C = −27.77 ± 0.04‰) and two IAEA-CH-6 sucrose standards (δ^13^C = −10.45 ± 0.03‰) in each run, as seen in Cotton, Sheldon & Strömberg (2012) study. Reproducibility of replicate analyses was better than 0.3‰.

To test whether other climate variables confounded the relationship between δ^13^C_atm_ and δ^13^C_leaf_, we calculated carbon isotope fractionation values (Δ_leaf_) for each specimen using known yearly δ^13^C_atm_ values (Eq. (1)). We derived global δ^13^C_atm_ values from direct (Rubino et al., 2013) and interpolated ice and firn core measurements according to the year of sampling (White, Vaughn & Michel, 2015). This does not account for ecosystem or microbiome-level deviations in δ^13^C_atm_, but does account for the greater source isotopic value. Δ_leaf_ values made it possible to de-couple the human-driven relationship between increased [CO_2_] and decreased δ^13^C_leaf_ values (the Suess Effect; Keeling, 1979) because they were calculated by isolating leaf isotope ratios from the atmospheric isotopic signal.

For each specimen locality, environmental data expected to affect δ^13^C_leaf_ values (MAP, MAT, and maximum summer temperature; Farquhar, Ehleringer & Hubick, 1989) were derived from global databases using exact latitude and longitude coordinates of specimen origin (PRISM Climate Group, 2004; Fick & Hijmans, 2017; Government of Canada, 2018). All contiguous United States data was compiled from Oregon State University’s PRISM database, which interpolates data from local weather stations at a resolution of four km (PRISM Climate Group, 2004). δ^13^C_leaf_ values were also compared with δ^13^C_atm_ and [CO_2_] values at collection times, as retrieved from NOAA databases documenting values found at Mauna Loa Observatory in Hawaii (White, Vaughn & Michel, 2015) and measured on an isotope-ratio mass spectrometer at the institute of arctic and alpine research (INSTAAR) in the University of Colorado, Boulder. δ^13^C_leaf_ values for which δ^13^C_atm_ values were unavailable were not included in Δ_leaf_ calculations or comparisons to climate variables (Table S3). While Δ_leaf_ values combine δ^13^C values measured in this experiment on the CRDS with δ^13^C_atm_ values measured on the IRMS at INSTAAR, we did not have access to individual errors for δ^13^C_atm_ and could not propagate the error. Therefore, we used our reproducibility error of 0.3‰, which is larger than the expected error for the IRMS (0.1‰), to be conservative.

Five-point moving averages of δ^13^C_leaf_ values were calculated to eliminate random noise caused by estimating older specimens’ exact collection dates (a result of long collecting expeditions and limited recording resources). We regressed isotope values against climate variables (MAT, MAP, δ^13^C_atm_ and annual [CO_2_]) to examine potential drivers of the δ^13^C_leaf_ values. We calculated the best-fit line using linear least squares regression to minimize the average distance between modeled *y*-values and actual *y*-values (δ^13^C_leaf_ and Δ_leaf_) and calculated coefficients of determination. We used *R*^2^ to determine predictive relationship between the given *x*-variable and *y*-variable. Additionally, we calculated *p*-values using the *F*-test to determine the chance of null hypothesis (*p*-value > 0.05).

The map of sampling location was created using R version 3.5.0 (R Core Team, 2014), and the *ggplot2* (Wickham, 2016) and *maps* (*v3.3.0*, Becker & Wilks, 2018) packages. The full code is available in the Supplemental Files.

## Results

Values of δ^13^C_leaf_ ranged from −21.92‰ (collected in 1899) to −28.51‰ (collected in 2017), with a mean of −25.05 ± 1.32‰ (standard deviation; Table S2). Δ_leaf_ values ranged from 15.11‰ to 20.97‰ (mean: 17.93 ± 1.11‰ standard deviation). Minimum, maximum, and mean values for climate variables are shown in Table S2. All data can be found in Table S3.

### Δ_leaf_ vs. MAT, maximum summer temperature, latitude, seasonality, and MAP

**Figure 2:**
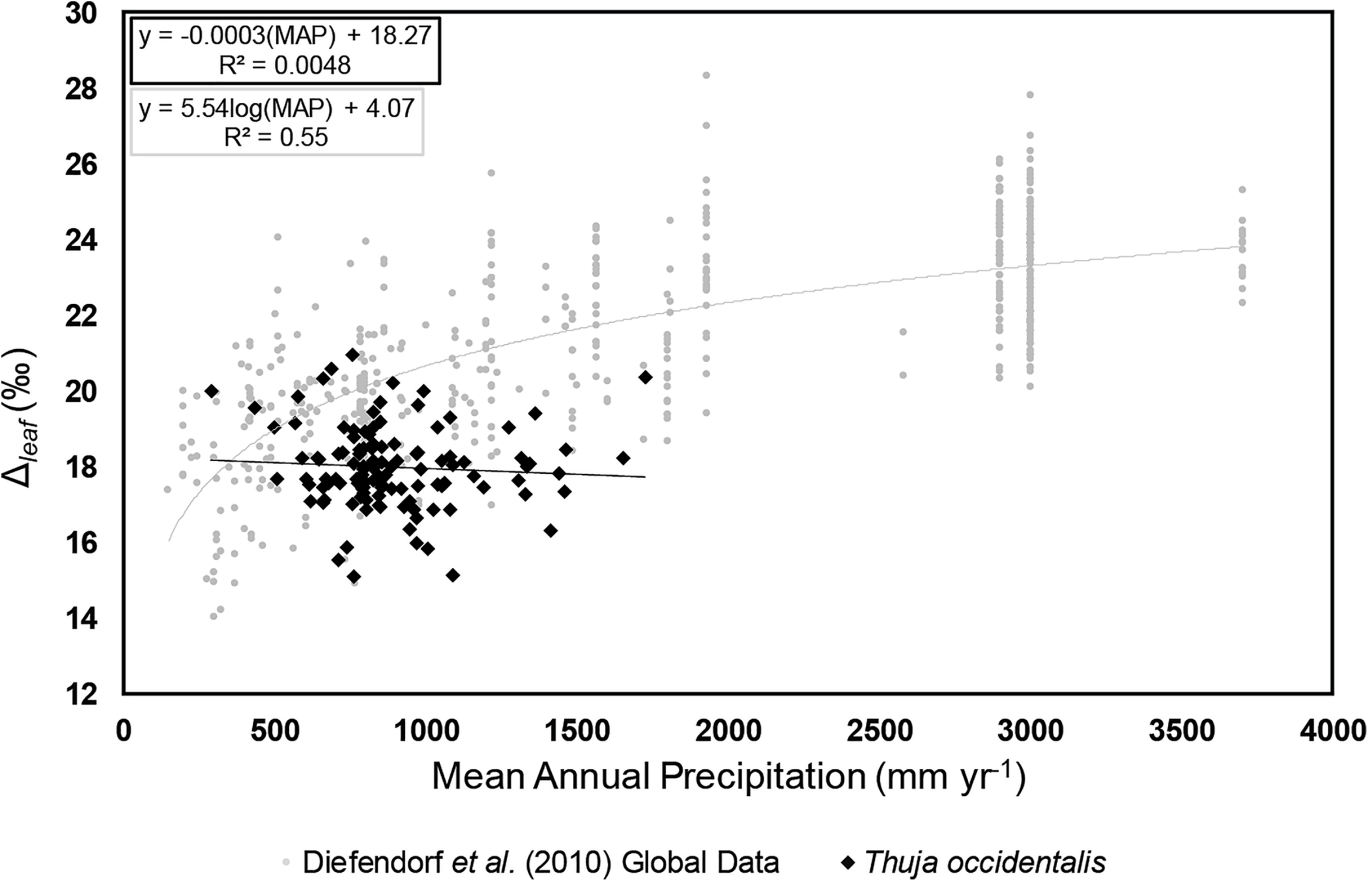
Carbon isotope fractionation values (Δ_leaf_) vs. mean annual precipitation for *Thuja occidentalis*. Δ_leaf_ vs. mean annual precipitation for *Thuja occidentalis* (black filled diamonds; Δ_leaf_ = −0.0003(MAP) + 18.75, *R*^2^ < 0.01). Data are compared with Diefendorf et al.’s (2010) global study (gray circles; Δ_leaf_ = 5.54(logMAP) + 4.07, *R*^2^ = 0.55), showing C_3_ specimens growing from 147 to 3,700 mm yr^−1^. Error bars along the *y*-axis represent the ±0.3‰ replicate reproducibility of standards.

There was no relationship between Δ_leaf_ values of *T. occidentalis* and MAT (Fig. S1A; *R*^2^ = 0.0152, *p*-value = 0.19), nor between Δ_leaf_ of *T. occidentalis* and maximum summer temperature (Fig. S1B; *R*^2^ = 0.0051, *p*-value = 0.51) in the temperature range listed in Table S2. No relationship was found between Δ_leaf_ and latitude (Fig. S1C; *R*^2^ = 0.0057, *p*-value = 0.42). Additionally, no relationship was found using a multivariate linear regression approach to combine codependent variables: MAT and latitude (*R*^2^ = 0.002, *p*-values = 0.92, 0.79 respectively). There was no relationship between Δ_leaf_ of *T. occidentalis* and MAP (Fig. 2; Fig. S1D; *R*^2^ = 0.0138, *p*-value = 0.21), nor was there a relationship between Δ_leaf_ of *T. occidentalis* and elevation (Fig. S4A; *R*^2^ = 0.0138, *p*-value = 0.55).

There was no relationship between Δ_leaf_ of *T. occidentalis* and month of collection. *T*-test results showed that the mean residual Δ_leaf_ values for each season were not significantly different (Spring: 0.19, Summer: −0.13, Fall: −0.01, Winter: −0.20; Fig. S5A; Month-by-month *R*^2^ = 0.0057; Fig. S5B).

### %C, %N and C:N ratios

**Figure 3:**
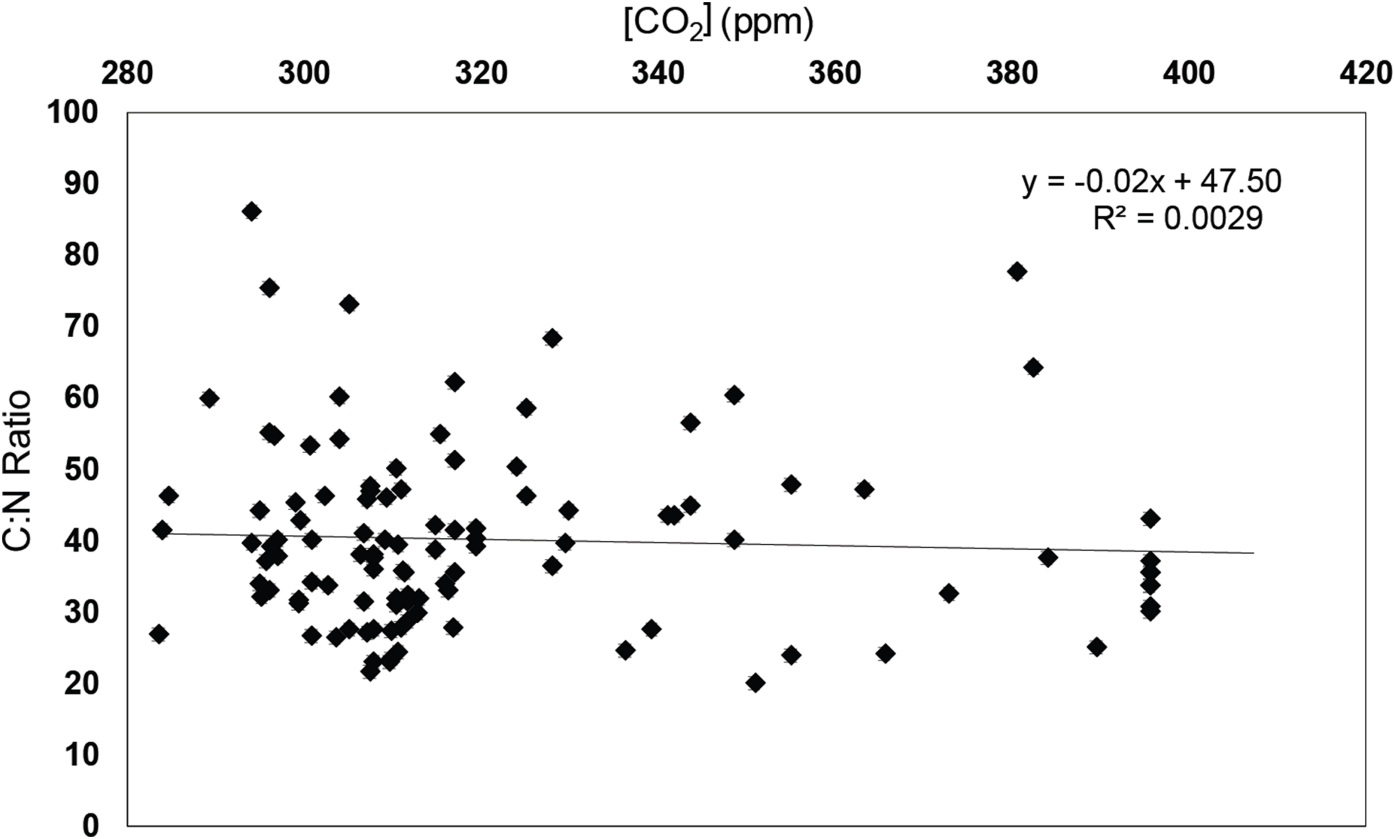
C:N ratios of specimens vs. [CO_2_]. C:N ratios of specimens vs. [CO_2_] (ppm) from Pre-Industrial values of 280–410 ppm. Error bars are associated with the 0.9% replicate reproducibility of standards.

Values of %C ranged from 30.56 weight % to 61.44 weight % (±7.90%), with a dataset average of 48.86 %C. %N values ranged from 0.55 weight % to 2.28 weight %, with a dataset average of 1.31% (±0.21%). C:N ratios ranged from 20.1:1 (due to high %N) to 86.2:1 with an average C:N value of 40.7:1 (±7.9 C:N). There was no relationship between C:N ratios and time, δ^13^C_leaf_, Δ_leaf_, or [CO_2_] (*R*^2^ = 0.0227, *p*-value = 0.25; *R*^2^ = 0.0297, *p*-value = 0.68; *R*^2^ = 0.0157, *p*-value = 0.94; and *R*^2^ = 0.0029, *p*-value = 0.18, respectively; Fig. 3; Figs. S2A–S2C).

### The atmosphere: δ^13^C_leaf_ and Δ_leaf_ vs. [CO_2_]

**Figure 4:**
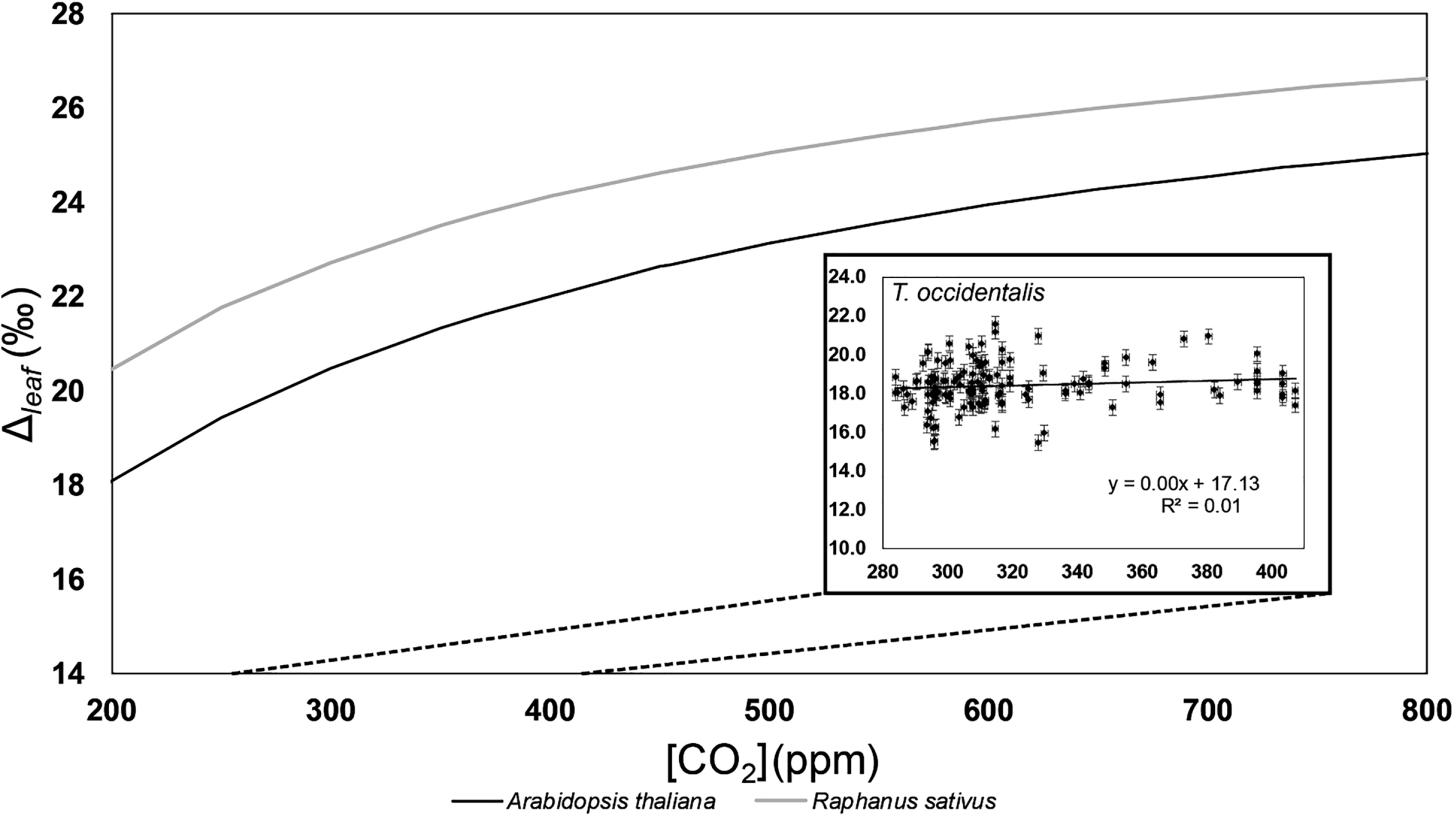
Carbon isotope fractionation values (Δ_leaf_) vs. [CO_2_] for *Thuja* and growth chamber dicots. Δ_leaf_ vs. [CO_2_] for *Thuja* and dicots. Shown are *Thuja occidentalis* (black empty triangles; Δ_leaf_ = 0.0029([CO_2_]) + 17.04, *R*^2^ = 0.0085) and Schubert & Jahren’s (2012) growth chamber studies with replicates of *Raphanus sativus* and *Arabidopsis thaliana* (black and grey smooth lines). Schubert & Jahren’s data shows C_3_ specimens growing at 15 levels of [CO_2_] from 370 to 4,200 ppm.

There was a linear relationship between δ^13^C_leaf_ and [CO_2_] (Fig. S3; *R*^2^ = 0.61, *p*-value < 0.001), but there was no relationship between Δ_leaf_ and [CO_2_] (Fig. 4; Fig. S4; *R*^2^ = 0.0059, *p*-value = 0.38). Because Δ_leaf_ stayed constant, with simultaneous changes in δ^13^C_leaf_ and δ^13^C_atm_, we can determine that changing δ^13^C_leaf_ with increased CO_2_ was an effect of the changing isotopic composition of atmospheric CO_2_ (δ^13^C_atm_) and was not related to [CO_2_].

### δ^13^C_atm_ and δ^13^C_leaf_

**Figure 5:**
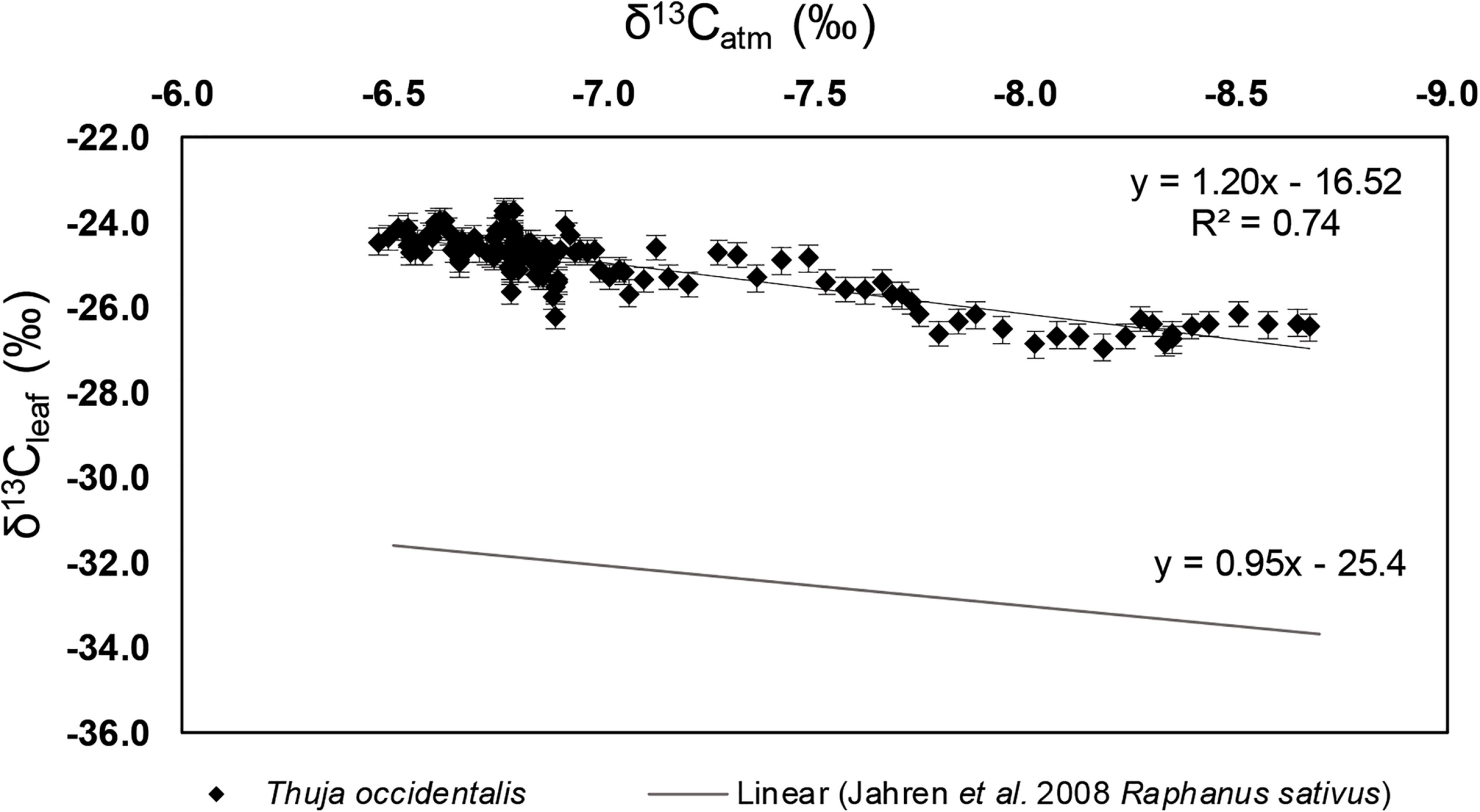
δ^13^C_leaf_ vs. δ^13^C_atm_ values of *Thuja occidentalis*. There is a linear relationship between δ^13^C_leaf_ and δ^13^C_atm_ values (‰) of *T. occidentalis*, defined by δ^13^C_leaf_ = 1.20 (±0.06 standard error) * (δ^13^C_atm_) − 16.52(±0.44 standard error) with an *R*^2^ value of 0.74 and a *p*-value <0.001. Error bars along the *y*-axis represent the ±0.3‰ replicate reproducibility of standards, error bars along the *x*-axis represent the ±0.1‰ replicate reproducibility associated with the isotope-ratio mass spectrometer at the Institute of Arctic and Alpine Research (INSTAAR) in Boulder, Colorado. Data points represent specimens collected for this study. The regression line below is derived from Jahren, Arens & Harbenson (2008), which used *Raphanus sativus* grown under elevated [CO_2_] within growth chambers to look at the relationship between δ^13^C_leaf_ and δ^13^C_atm_ values.

There was a relationship between δ^13^C_leaf_ and δ^13^C_atm_ (Fig. 5; *R*^2^ = 0.74, *p*-value < 0.001) as represented by Eq. (2). The *y*-intercept (−16.52) represents the average offset between δ^13^C_atm_ and δ^13^C_leaf_, otherwise expressed as the fractionation value, Δ_leaf_. The slope of 1.20 (with 95% confidence intervals between 1.07 and 1.32) further indicates relatively little impact of other environmental variables on leaf fractionation from atmospheric CO_2_. This relationship was compared to that as extrapolated from the regression found in Jahren, Arens & Harbenson (2008; Eq. (3); Fig. 5) study using *Raphanus sativus* in growth chamber experiments.

(2) $${{\rm{\delta }}^{13}}{{\rm{C}}_{{\rm{leaf}}}} = 1.20\,\left( { \pm 0.06} \right)\, \times {{\rm{\delta }}^{13}}{{\rm{C}}_{{\rm{atm}}}} - 16.52\left( { \pm 0.44} \right)$$

(3) $${{\rm{\delta }}^{13}}{{\rm{C}}_{{\rm{leaf}}}} = 0.95\, \times {{\rm{\delta }}^{13}}{{\rm{C}}_{{\rm{atm}}}} - 25.4$$

## Discussion

### Potentially complicating factors

Herbarium specimens are a useful way to look in high-resolution on this time scale, but were not necessarily consistently sampled between expeditions and years. Thus some factors such as maturity and height of tree, which have been shown to relate to carbon isotope discrimination (Brienen et al., 2017), are not specifically accounted for here. However, Brienen et al. (2017) found that height was not a significant factor in δ^13^C_leaf_ values of the gymnosperm used (*P. sylvestris*), in contrast to broadleaf species (*Quercus robur*, *Fagus sylvatica*, *Cedrela odorata*) where there was an influence. Additionally, regional variations in δ^13^C_atm_ resulting from proximity to respiring soil could provide a different baseline δ^13^C_atm_ value than the global one used based upon Mauna Loa Observatory’s gas samples (Wehr & Saleska, 2015). While we were unable to control exactly where on the tree each sample was taken from nor the ecosystem-specific parameters that might influence δ^13^C_atm_, each herbarium record includes notes on the sampling location, often including approximate maturity of the tree and height sampled from. Additionally, as expeditions are done without heavy machinery, it is likely that our specimens were sampled from approximately the height of a human, which is well out of the range of isotopic influence from soil respired CO_2_ (Bazzaz & Williams, 1991). Finally, differences in δ^13^C_leaf_ values differ depending on where on the leaf isotopes are sampled from (Gao et al., 2015). We controlled for this effect by homogenizing several entire leaves per sample and running duplicate isotope analyses, which all came within machine error (0.3‰) of one another.

Complex nitrogen dynamics, as well as those of other macro- and micro-nutrients, may play a role in vital effects that relate ultimately to carbon assimilation and/or carbon isotope fractionation, but these changes are very difficult and complicated to reconstruct in the geologic record and thus were not investigated in this study (Godfrey & Glass, 2011). It is not possible to understand shifts in regional nitrogen availability fully even within the historical record: without soil cores collected and preserved from the same sites and times of leaf collection, it is not straightforward to consider anything other than nitrogen content. Given that no one collected soils at each historical site, we cannot address changes in nitrogen dynamics quantitatively. Furthermore, for geologic applications fossil leaves and corresponding paleosols (fossil soils) are not typically preserved together. In order to account for the potential range in nitrogen dynamics, we focus here on using many specimens from across a wide landscape of different land-uses and thus a wide range of potential nitrogen dynamics, but under known [CO_2_] conditions. To account for historical and geological limitations of reconstructing the nitrogen cycle, this study focuses instead on the relationship between systemic changes in C:N ratios in relation to δ^13^C_leaf_, δ^13^C_atm_, or as indication of changes in carbon dynamics.

### Δ_leaf_, temperature, latitude and seasonality

There were a number of climate variables that we did not expect to have a correlative relationship with Δ_leaf_, but we addressed to ensure that they were not confounding variables (MAT, maximum summer temperature, latitude, seasonality). Due to the consistent internal temperature and lack of relationship between Δ_leaf_ to MAT in previous studies, we expected no relationship between Δ_leaf_ and MAT nor maximum summer temperature. As we expected, MAT showed no significant relationship with Δ_leaf_ values neither in meta-analyses, growth chamber experiments (Diefendorf et al., 2010; Schubert & Jahren, 2012) nor this study (Fig. S1A).

Because MAT and latitude are inherently related, we expected Δ_leaf_ values to relate to latitude in the same way as when compared to MAT. As predicted, there was no relationship between Δ_leaf_ of *T. occidentalis* and latitude. In order to ensure that these two codependent variables were treated as such, we ran a multiple linear regression, which gave a similarly low coefficient of determination (*R*^2^ = 0.002) comparing actual Δ_leaf_ values with Δ_leaf_ values predicted using this regression. Thus, we are confident that both treated as independent and co-dependent variables, MAT and latitude do not play a role in variance of Δ_leaf_ values.

Additionally, we took into account variation in time of year specimen was collected and found no significant relationship between season and Δ_leaf_ (Figs. S5A and S5B). This is likely because *T. occidentalis* is not deciduous and does not shed its leaves annually, thus, once homogenized, the isotopic composition of the leaf is representative of average discrimination during the leaf’s exposure.

### Δ_leaf_ and [CO_2_]

In this study we found no relationship between Δ_leaf_ and [CO_2_] from 280 to 410 ppm (Fig. 4; Fig. S4). It is possible that the relationship observed by Schubert & Jahren (2012) exists only for idealized controlled growth chamber conditions and not in natural environments (Lomax et al., 2019). Alternatively, gymnosperms may respond more slowly than angiosperms to increases in [CO_2_] due to their longer average lifespans and lack of senescence (Brodribb, Pittermann & Coomes, 2012). The changes in carbon assimilation as represented by increased carbon fractionation under short-term, ideal growth chamber conditions cannot be used to predict biological response to rapid changes in [CO_2_]. In other words, plants, especially slow-growing woody plants, may not successfully adapt to anthropogenic changes of the present and future (Jump & Peñuelas, 2005).

### Δ_leaf_ and mean annual precipitation

When Δ_leaf_ values of *T. occidentalis* were compared with MAP, Diefendorf et al.’s (2010) previously established relationship did not hold (Fig. 2), especially in low precipitation regimes (<1,000 mm yr^−1^) where the change in Diefendorf et al.’s Δ_leaf_ values was most sensitive to changes in MAP. One explanation for the lack of relationship between Δ_leaf_ of *T. occidentalis* and MAP is that this relationship breaks down on the single-species, or even plant functional type, level. In the aforementioned meta-analyses, plant functional type, species, and region were not controlled. Δ_leaf_ values may be inherent to specific biomes but may not be representative of a general trend of any given plant or plant type to MAP. It is possible that the relationships seen in the meta-analyses by Diefendorf et al. (2010) and Kohn (2010) instead represent an array of taxon-specific constant isotopic values that collectively show a meta-relationship. This experiment could be further explored by performing the same experiment in natural settings across different biomes and different plant functional types. Assuming that Δ_leaf_ values are indicative of water use (Givnish, 1979; Farquhar, Ehleringer & Hubick, 1989), this lack of relationship may also mean that plants with specific water use efficiencies and representative Δ_leaf_ values are generally located in areas where they are not living in conditions that are stressed for water given their evolutionary adaptations. Geologically, this could mean that the presence of a particular taxon in fossil localities could provide a quantitative estimate for range of MAP, which could allow more specificity of paleoclimate regimes based on macrofossils (Nearest Living Relative and/Coexistence Approach; Mosbrugger & Utescher, 1997; Mosbrugger, 2009). In terms of future climate, this is indication that the chemistry of C_3_ plants may not respond to regional changes as previously thought. This is of particular concern because the velocity of climate change, especially for continued high emission rate scenarios, is substantially faster than trees will be able to adapt to (Loarie et al., 2009; Diffenbaugh & Field, 2013).

It is also possible that the predicted relationship between Δ_leaf_ and MAP is present for plants that are more responsive to their environment and/or have less extensive roots (to access deeper water sources), and thus the signal seen by Diefendorf et al. (2010) is a result of incorporating sensitive plants. Indeed, other studies using rapidly growing, highly sensitive herbaceous angiosperms have found a relationship between water treatment and carbon isotope discrimination (Lomax et al., 2019). However, the fossil record is biased toward preserving less sensitive, often woody plants due to preservation potential as well as presence within the fossil record (Looy et al., 2014); therefore, the utility of relationships based on highly sensitive plants may be muted in the fossil record.

Another explanation is that MAP is not an appropriate metric for measuring plant-available water, and while Δ_leaf_ is still a measure of water use efficiency and this value is dynamic over conditions, snowmelt volumes and/or soil water—as driven by soil porosity and other factors—are better indicators of plant-available water. Further investigations using these variables will better constrain which water-related variables affect Δ_leaf_ values of leaf tissues. The weak R^2^ value between Δ_leaf_ of *T. occidentalis* and MAP (0.0138) means that Δ_leaf_ of *T. occidentalis* cannot be used to reconstruct paleo-MAP using the relationship determined by Diefendorf et al. (2010). Additional single-species experiments, particularly within angiosperms, should be conducted to look for correlations between Δ_leaf_ and MAP to test whether the lack of relationship is due to a difference inherent to gymnosperms.

### Carbon biomass (%C) and elemental leaf chemistry (%N, C:N ratios) as related to climate variables

In addition to the response of δ^13^C_leaf_ values to climate variables, %C alone has been shown to respond directly to elevated [CO_2_]. *C_i_*/*C_a_* ratios (the ratio of internal [CO_2_] to atmospheric [CO_2_]) of old growth *T. occidentalis* trees along Lac Duparquet, Quebec, increased under anthropogenic CO_2_ fertilization, indicating tree response to enhanced CO_2_ (Giguère-Croteau et al., 2019). This increase in tree productivity was demonstrated in the results of free-air concentration enrichment (FACE) experiments as well; in northern USA mid-latitude forests with loblolly pines (*P. taeda*), FACE experiment results indicated that elevated CO_2_ induced increased carbon assimilation, resulting in increased carbon biomass, in woody tissues and increased %C of foliar storage as compared to trees grown under ambient CO_2_ (Oren et al., 2001; Ainsworth & Long, 2005; Talhelm et al., 2013). Preliminary work in herbarium leaves found that increased [CO_2_] related to Industrialization resulted in an increase in foliar %C with no change in %N (as source of N remains constant), and thus increased C:N ratios in some species (Mervenne, 2015). In order to contextualize changes in δ^13^C_leaf_, this study examined coeval trends in leaf chemistry through elemental analysis of C and N (using N as a comparison point to see whether %C changes significantly with time).

These FACE experiments also showed that when run over longer time scales, trees reached a point of CO_2_ acclimation and stopped increased carbon assimilation under enhanced CO_2_; thus, predicted shifts in tree C-uptake may be short-lived, a pattern that will be inevitably discernable in a long-term study incorporating pre-Industrial leaf tissues through the present (Nowak, Ellsworth & Smith, 2004). Based on FACE experiments, we expected %C to have increased in leaves sampled from the early 1800s to the present, though we might see the rate of increase slow with time. However, we saw no relationship between %C, nor C:N ratios and time nor increase in [CO_2_].

In fact, *T. occidentalis* specimens collected between 1804 and 2017 did not show changes in assimilation rates due to elevated CO_2_. C:N values of *T. occidentalis* showed no response to changes over time (with increased [CO_2_]) or with atmospheric isotopic value (Figs. S2A and S2B). Though other organs in previous experiments responded to [CO_2_], leaves, which are instrumental in the photosynthetic process as they are the organs directly in-taking atmospheric CO_2_, do not. A better understanding of all plant organ behavior is imperative to defining and quantifying potential carbon sinks or plant chemistry responses to global change (Goodale et al., 2002).

### δ^13^C_leaf_ and δ^13^C_atm_

Strong relationships have been found between above ground tissue and δ^13^C_atm_ values (*p* < 0.001; Jahren, Arens & Harbenson, 2008; Fig. 5), and this study provides a higher resolution look at the relationship between δ^13^C_leaf_ and δ^13^C_atm_ in a long-lived species within a natural system. In this initial natural experiment, the δ^13^C_leaf_ of *T. occidentalis* tracked changes in δ^13^C_atm_ (*R*^2^ = 0.74, *p*-value < 0.0001), mostly unencumbered by other climate factors. The slope for the linear relationship between δ^13^C_leaf_ and δ^13^C_atm_ is close to, but not exactly, 1, likely because the rate of change for δ^13^C_atm_ has not been linear, and acceleration in the change of δ^13^C_atm_ may not have been recorded immediately. Additionally, while there is no statistically significant relationship between any of the climate variables we tested and Δ_leaf_, it is unlikely that climate variables, especially in aggregate, play no role in carbon isotope discrimination within this species. Because δ^13^C_leaf_ showed a strong coefficient of determination with δ^13^C_atm_, and no climate variables showed significant relationships with Δ_leaf_ values, we can assume that δ^13^C_leaf_ values of modern *T. occidentalis* are strongly affected by δ^13^C_atm_ values. Additional work must be done to evaluate error in paleo uses of δ^13^C_leaf_ values of *T. occidentalis*, and future experiments should recreate more geologically reasonable conditions and climate changes (independent of anthropogenic factors). The relationship between δ^13^C_leaf_ and δ^13^C_atm_ values has implications for paleoclimate reconstructions of δ^13^C_atm_ as well as reconstructions of [CO_2_] (Cerling et al., 1991; Franks et al., 2014). We emphasize how important it is to identify the value of δ^13^C_atm_, such as in Tipple, Meyers & Pagani (2010) study, rather than just using the Pre-Industrial value of −6.5‰ (Cerling et al., 1991) because the δ^13^C_atm_ value has such a dramatic effect on the terrestrial part of the carbon cycle.

## Conclusions

Though δ^13^C_leaf_ and Δ_leaf_ values have been proposed as a proxy for [CO_2_] and MAP based on previous research, this natural-world, species-controlled study shows no indication of such relationships. Thus, the use of Δ_leaf_ values to reconstruct MAP and [CO_2_] in the fossil record without taxonomic identification should be reconsidered. The relationship between δ^13^C_leaf_ and δ^13^C_atm_ values is more informative, and may provide a new proxy (δ^13^C_leaf_ values of *Thuja*) for reconstructing paleo- δ^13^C_atm_ or may indicate a lag in plant adaptation to unprecedentedly rapid climate change. *Thuja* extends up to 100 million years back to the Late Cretaceous, which makes this relationship potentially useful throughout the Cenozoic and into the Mesozoic era (Berry, 1915).

While this study focuses on one single species, further work is needed to assess other taxa at the species, genus, and family levels to examine whether the relationship between δ^13^C_atm_ and δ^13^C_leaf_ is consistent, and furthermore, generalizable. δ^13^C_leaf_ values of individual fossil leaves (in particular of *Thuja* leaves) cannot be used to reconstruct paleo-MAP as proposed by Kohn (2010), but average δ^13^C_leaf_ values of sites, as recorded in bulk soil organic matter, may allow us to predict precipitation ranges. Aboveground δ^13^C_leaf_ is thought to translate directly into the isotopic value of soil carbon (δ^13^C_org_; Arens, Jahren & Amundson, 2000). Bulk soil organic matter (δ^13^C_org_) is the combination of δ^13^C of all decaying material from the ecosystem, with leaves especially abundant due to sheer volume. The average δ^13^C_leaf_ value of all trees found in a certain region will be found in the soil; therefore, soil δ^13^C_org_ values could be more reflective of particular precipitation at time of deposition than δ^13^C_leaf_ values. Further studies could evaluate the reliability of δ^13^C_org_ as a tool for MAP prediction and reconstruction.

This study implies constant carbon and nitrogen use and isotope fractionation relative to δ^13^C_atm_ by *T. occidentalis*. Due to the unprecedentedly rapid changes δ^13^C_atm_ and [CO_2_] throughout Industrialization, this lack of change in carbon assimilation patterns, despite previous studies using modern δ^13^C_leaf_ values to reconstruct [CO_2_], may indicate that modern systems are not appropriate analogues for many periods of the geologic record during which climate evolved more slowly. Modern climate change may be too rapid for plants to adapt, though more research should be done to evaluate whether this response is replicable in other species, genera, and plant functional types. It is possible that the pace of anthropogenic climate change makes modern relationships inappropriate analogues for paleoclimate.

## Supplemental Information

10.7717/peerj.7378/supp-1 Supplemental Information 1 Δ*_leaf_* values vs. climate variables. a) Δ*_leaf_* values vs. mean annual temperature (−5 to 14°C) for collected specimens (R^2^ = 0.0001). b) Δ*_leaf_* values vs. maximum summer (growing season) temperature (20 to 33°C) for collected specimens (R^2^ = 0.0005). c) Δ*_leaf_* values vs. latitude (43 to 63°N) for collected specimens (R^2^ = 0.0002). d) Δ*_leaf_* values vs. mean annual precipitation (288 to 1724 mm yr^−1^) for collected specimens (R^2^ = 0.0005).

10.7717/peerj.7378/supp-2 Supplemental Information 2 C:N vs. climate and temporal variables. C:N ratios as determined by weight %C and weight %N measured on a Costech Elemental Analyzer compared to a) time collected (year), b) δ^13^C_atm_ values (‰), c) δ^13^C_leaf_ values (‰). Error bars on the y-axis are associated with the 0.9% replicate reproducibility of standards. Error bars on the x-axis in c) are associated with the 0.3‰ replicate reproducibility of the Picarro CRDS standards.

10.7717/peerj.7378/supp-3 Supplemental Information 3 δ^13^C_leaf_ values vs. *p*CO_2_. Linear regression between δ^13^C_leaf_ values (‰) and [CO_2_] (283 to 407 ppm) for collected specimens (R^2^ = 0.61). Error bars along the y-axis represent the ±0.3‰ replicate reproducibility of standards.

10.7717/peerj.7378/supp-4 Supplemental Information 4 Δ*_leaf_* vs. elevation & *p*CO_2_. a) Linear regression between Δ*_leaf_* values vs. elevation (4 to 1617 m above sea level). Error bars along the y-axis represent the ±0.3‰ replicate reproducibility of standards b) Δ*_leaf_* and *p*CO_2_ (283 to 407 ppm) for collected specimens (R^2^ = 0.01). Error bars along the y-axis represent the ±0.3‰ replicate reproducibility of standards.

10.7717/peerj.7378/supp-5 Supplemental Information 5 δ^13^C_leaf_ and seasonality. a) Box and Whisker plot showing median (lines), mean and range of spring, summer, fall and winter residual values of δ^13^C_leaf_. T-tests assuming unequal variance comparing Spring vs. Summer, Fall, Winter, Summer vs. Fall, Winter, and Fall vs. Winter could not prove a null hypothesis; that the mean for all seasonal collections was significantly different. Seasons were categorized by mean monthly temperatures <°2C, from 2–15°C and rising, >15°C, and between 2–15°C and falling in the Great Lakes Region (centralized lower Peninsula Michigan), b) Scatter plot including residual from mean δ^13^C_leaf_ values as divided by month.

10.7717/peerj.7378/supp-6 Supplemental Information 6 Distribution of herbarium and botanical garden specimen data.

10.7717/peerj.7378/supp-7 Supplemental Information 7 The range and mean values for measured climate variables in this study. The minimum, maximum, and mean values for climate conditions for each specimen as obtained from NOAA ESRL Global Monitoring Division (2016), White *et al.* 2015, and PRISM Climate Group, as well as Government of Canada (Ed.). (2018, January 11).

10.7717/peerj.7378/supp-8 Supplemental Information 8 Raw Data. *Direct Mauna Loa measurements (NOAA ESRL Global Monitoring Division (2016).
**White et al. (2015)) and Petit et al. (2001) ice core records.
***: for United States Climate Data: PRISM Climate Group Oregon State University. (2017). Data Explorer: Time Series Values for Individual Locations. Retrieved from Northwest Alliance for Computational Science & Engineering database. For Canadian Climate Data: Government of Canada (Ed.). (2018, January 11). All specimens used for isotope analysis are stored in the University of Michigan’s Earth Systems Laboratory (Dr. Nathan Sheldon & Dr. Ingrid Hendy) within the University of Michigan Earth and Environmental Science Department.

10.7717/peerj.7378/supp-9 Supplemental Information 9 Figure 1 Code.

## Additional Information and Declarations

### Data Availability

The following information was supplied regarding data availability: Raw data is available in Table S3, which provides our raw isotope, elemental, and climate data. All specimens used in isotope analysis are stored in the University of Michigan’s Earth Systems Lab (of Dr. Ingrid Hendy and Dr. Nathan Sheldon) within the Earth and Environmental Science Department. The living (or former) trees sampled are located at the provided locations in Table S3 Column D, “Original tree location” (GPS columns J and K “Latitude” and “Longitude” respectively), and the residence of the herbaria collections is listed in Table S3 column E “Source.”

## References

[ref-1] Ainsworth EA, Long SP (2005). What we have learned from 15 years of free-air CO_2_ enrichment (FACE)? A meta-analytic review of the responses of photosynthesis, canopy properties, and plant production to rising CO_2_. New Phytologist.

[ref-2] Ainsworth EA, Rogers A (2007). The response of photosynthesis and stomatal conductance to rising [CO_2_]: mechanisms and environmental interactions. Plant, Cell & Environment.

[ref-5] Araus JL, Buxó R (1993). Changes in carbon isotope discrimination in grain cereals from the north-western Mediterranean Basin during the past seven millennia. Functional Plant Biology.

[ref-6] Arens NC, Jahren AH, Amundson R (2000). Can C_3_ plants faithfully record the carbon isotopic composition of atmospheric carbon dioxide?. Paleobiology.

[ref-7] Augustin L, Barbante C, Barnes PR, Barnola JM, Castellano E, Dreyfus G (2004). Eight glacial cycles from an Antarctic ice core. Nature.

[ref-8] Barnola JM, Raynaud D, Korotkevich YS, Lorius C (1987). Vostok ice core provides 160,000-year record of atmospheric CO_2_. Nature.

[ref-10] Bazzaz FA, Williams WE (1991). Atmospheric CO_2_ concentrations within a mixed forest: implications for seedling growth. Ecology.

[ref-11] Beerling DJ, Mattey DP, Chaloner WG (1993). Shifts in the δ^13^C composition of *Salix herbacea* L. leaves in response to spatial and temporal gradients of atmospheric CO_2_ concentration. Proceedings of the Royal Society of London. Series B: Biological Sciences.

[ref-12] Beerling DJ, Royer DL (2002). Fossil plants as indicators of the Phanerozoic global carbon cycle. Annual Review of Earth and Planetary Sciences.

[ref-13] Berry EW (1915). The age of the Cretaceous flora of southern New York and New England. Journal of Geology.

[ref-14] Boutton TW (1991). Stable carbon isotope ratios of natural materials: II. Atmospheric, terrestrial, marine, and freshwater environments. Carbon Isotope Techniques.

[ref-15] Brienen RJW, Gloor E, Clerici S, Newton R, Arppe L, Boom A, Bottrell S, Callaghan M, Heaton T, Helama S, Helle G, Leng MJ, Mielikainen K, Oinonen M, Timonen M (2017). Tree height strongly affects estimates of water-use efficiency responses to climate and CO_2_ using isotopes. Nature Communications.

[ref-16] Brodribb TJ, Pittermann J, Coomes DA (2012). Elegance versus speed: examining the competition between conifer and angiosperm trees. International Journal of Plant Sciences.

[ref-17] Cerling TE (1992). Use of carbon isotopes in paleosols as an indicator of the *p*(CO_2_) of the paleo atmosphere. Global Biogeochemical Cycles.

[ref-18] Cerling TE, Solomen DK, Quade J, Bowman JR (1991). On the isotopic composition of carbon in soil carbon dioxide. Geochimica et Cosmochimica Acta.

[ref-20] Cotton JM, Sheldon ND, Strömberg CAE (2012). High-resolution isotopic record of C_4_ photosynthesis in a Miocene grassland. Palaeogeography, Palaeoclimatology, Palaeoecology.

[ref-21] Deines P, Schidlowski M, Golubic S, Kimberley MM, McKirdy DM, Trudinger PA (1992). Mantle carbon: concentration, mode of occurrence, and isotopic composition. Early Organic Evolution: Implications for Mineral and Energy Resources.

[ref-22] Diefendorf AF, Leslie AB, Wing SL (2015). Leaf wax composition and carbon isotopes vary among major conifer groups. Geochimica et Cosmochimica Acta.

[ref-23] Diefendorf AF, Mueller KE, Wing SL, Koch PL, Freeman KH (2010). Global patterns in leaf ^13^C discrimination and implications for studies of past and future climate. Proceedings of the National Academy of Sciences of the United States of America.

[ref-24] Diffenbaugh NS, Field CB (2013). Changes in ecologically critical terrestrial climate conditions. Science.

[ref-26] Eckenwalder JE (2009). Conifers of the world: the complete reference.

[ref-28] Ehleringer JR, Phillips SL, Comstock JP (1992). Seasonal variation in the carbon isotopic composition of desert plants. Functional Ecology.

[ref-29] Elsig J, Schmitt J, Leuenberger D, Schneider R, Eyer M, Leuenberger M, Stocker TF (2009). Stable isotope constraints on Holocene carbon cycle changes from an Antarctic ice core. Nature.

[ref-30] Farquhar GD, Ehleringer JR, Hubick KT (1989). Carbon isotope discrimination and photosynthesis. Annual Review of Plant Biology and Plant Molecular Biology.

[ref-31] Farquhar GD, Sharkey TD (1982). Stomatal conductance and photosynthesis. Annual Review of Plant Physiology.

[ref-32] Feng X (1999). Trends in intrinsic water-use efficiency of natural trees for the past 100–200 years: a response to atmospheric CO2 concentration. Geochimica et Cosmochimica Acta.

[ref-33] Fick SE, Hijmans RJ (2017). Wordclim 2: new 1-km spatial resolution climate surfaces for global land areas. International Journal of Climatology.

[ref-35] Franks PJ, Royer DL, Beerling DJ, Van de Water PK, Cantrill DJ, Barbour MM, Berry JA (2014). New constraints on atmospheric CO_2_ concentration for the Phanerozoic. Geophysical Research Letters.

[ref-111] Gao L, Guimond J, Thomas E, Huang Y (2015). Major trends in leaf wax abundance, δ^2^H and δ^13^C values along leaf venation in five species of C_3_ plants: physiological and geochemical implications. Organic Geochemistry.

[ref-38] Giguère-Croteau C, Boucher E, Bergeron Y, Girardin MP, Drobyshev I, Silva LCR, Helie JF, Garneau M (2019). North America’s oldest boreal trees are more efficient water users due to increased [CO_2_], but do not grow faster. Proceedings of the National Academy of Sciences of the United States of America.

[ref-39] Givnish T, Solbrig OT, Jain S, Johnson GB, Raven PH (1979). On the adaptive significance of leaf form. Topics in Plant Population Biology.

[ref-40] Givnish T (2002). Adaptive significance of evergreen vs. deciduous leaves: solving the triple paradox. Silva Fennica.

[ref-41] Godfrey LV, Glass JB (2011). The geochemical record of the ancient nitrogen cycle, nitrogen isotopes, and metal cofactors. Methods in Enzymology.

[ref-42] Goodale CL, Apps MJ, Birsey RA, Field CB, Heath LS, Houghton RA, Jenkins JC, Kohlmaier GH, Kurz W, Liu S, Nabuurs GJ, Nilsson S, Schvidenko AZ (2002). Forest carbon sinks in the northern hemisphere. Ecological Applications.

[ref-43] Government of Canada (2018). 1981–2010 Climate Normals & Averages. http:/climate.weather.gc.ca/climate_normals.

[ref-44] Gröcke DR (2002). The carbon isotope composition of ancient CO_2_ based on higher-plant organic matter. Philosophical Transactions of the Royal Society of London. Series A: Mathematical, Physical and Engineering Sciences.

[ref-45] Helliker BH, Richter SL (2008). Subtropical to boreal convergence of tree-leaf temperatures. Nature.

[ref-46] IPCC (2014). Intergovernmental panel on climate change guidelines.

[ref-47] Jahren AH, Arens NC, Harbenson SA (2008). Prediction of atmospheric δ^13^CO_2_ using fossil plant tissues. Reviews of Geophysics.

[ref-48] Jump AS, Peñuelas J (2005). Running to stand still: adaptation and the response of plants to rapid climate change. Ecology Letters.

[ref-49] Keeling CD (1979). The Suess effect: ^13^Carbon-^14^Carbon interrelations. Environment International.

[ref-50] Keeling CD, Piper SC, Bascatow RB, Wahlen M, Whorf TP, Heimann M, Meijer HA, Ehleringer JR, Cerling T, Dearing MD (2005). Exchanges of atmospheric CO_2_ and ^13^CO_2_ with the terrestrial biosphere and oceans from 1978-2000: observations and carbon cycle implications. A History of Atmospheric CO_2_ and its Effects on Plants, Animals, and Ecosystems.

[ref-51] Keeling CD, Whorf TP (2004). Atmospheric CO_2_ from continuous air samples at Mauna Loa observatory.

[ref-53] Kohn MJ (2010). Carbon isotope compositions of terrestrial C3 plants as indicators of (paleo) ecology and (paleo) climate. Proceedings of the National Academy of Sciences of the United States of America.

[ref-55] Korner C, Farquhar GD, Wong SC (1991). Carbon isotope discrimination by plants follows latitudinal and altitudinal trends. Oecologia.

[ref-109] LePage BA (2003). A new species of Thuja (Cupressaceae) from the Late Cretaceous of Alaska: implications of being evergreen in a polar environment. American Journal of Botany.

[ref-56] Loarie SR, Duffy PB, Hamilton H, Asner GP, Field CB, Ackerly DD (2009). The velocity of climate change. Nature.

[ref-57] Lomax BH, Lake JA, Leng MJ, Jardine PE (2019). An experimental evaluation of the use of Δ^13^C as a proxy for palaeoatmospheric CO_2_. Geochimica et Cosmochimica Acta.

[ref-58] Looy C, Kerp H, Duijnstee I, DiMichele B (2014). The late Paleozoic ecological-evolutionary laboratory, a land-plant fossil record perspective. Sedimentary Record.

[ref-59] Lowdon J, Dyck W (1974). Seasonal variations in the isotope ratios of carbon in maple leaves and other plants. Canadian Journal of Earth Sciences.

[ref-61] Mårtensson LM, Carlsson G, Prade T, Kørup K, Lærke PE, Jensen ES (2017). Water use efficiency and shoot biomass production under water limitation is negatively correlated to the discrimination against ^13^C in the C_3_ grasses *Dactylis glomerata, Festuca arundinacea* and *Phalaris arundinacea*. Plant Physiology and Biochemistry.

[ref-62] Medlyn BE, Barton CVM, Rey A, Roberntz P, Sigurdsson BD, Strassemeyer J, Wang K, Curtis PS, Jarvis PG, Broadmeadow MSJ, Ceulemans R, De Angelis P, Forstreuter M, Freeman M, Jackson SB, Kellomaki S, Laitat E (2001). Stomatal conductance of European forest species after long-term exposure to elevated [CO_2_]: a synthesis of experimental data. New Phytologist.

[ref-63] Mervenne C (2015). Isotope ecology of temperate conifers.

[ref-64] Mirza MMQ (2003). Climate change and extreme weather events: can developing countries adapt?. Climate Policy.

[ref-65] Mosbrugger V, Gornitz V (2009). Nearest-living-relative method. Encyclopedia of Paleoclimateology and Ancient Environments.

[ref-66] Mosbrugger V, Utescher T (1997). The coexistence approach—a method for quantitative reconstructions of Tertiary terrestrial paleaeoclimate data using plant fossils. Palaeogeography, Palaeoclimatology, Palaeoecology.

[ref-68] Nowak RS, Ellsworth DS, Smith SD (2004). Functional responses of plants to elevated atmospheric CO_2_- do photosynthetic and productivity data from FACE experiments support early predictions?. New Phytologist.

[ref-70] O’Leary MH (1993). Biochemical basis of carbon isotope fractionation. Stable Isotopes and Plant Carbon-Water Relations.

[ref-72] Oren R, Ellsworth DS, Johnsen KH, Phillips N, Ewers BE, Maier C, Schafer KR, McCarthy H, Hendrey G, McNulty SG, Katul GG (2001). Soil fertility limits carbon sequestration by forest ecosystems in a CO_2_-enriched atmosphere. Nature.

[ref-9] Original S code by Richard A. Becker, Allan R. Wilks. R version by Ray Brownrigg. Enhancements by Thomas P Minka and Alex Deckmyn (2018). https://CRAN.R-project.org/package=maps.

[ref-73] Pease VA (1917). Duration of leaves in evergreens. American Journal of Botany.

[ref-74] Pedicino LC, Leavitt SW, Betancourt JL, Van de Water PK (2002). Historical variations in δ^13^C_leaf_ of herbarium specimens in the southwestern U. S. Western North American Naturalist.

[ref-75] Peñuelas J, Azcón-Bieto J (1992). Changes in leaf Δ^13^C of herbarium plant species during the last 3 centuries of CO_2_ increase. Plant, Cell & Environment.

[ref-76] Petit JR, Jouzel J, Raynaud D, Barkov NI, Barnola JM, Basile I, Bender M, Chappellaz J, Davis M, Delaygue G, Delmotte M, Kotlyakov M, Legrand M, Lipenkov VY, Lorius C, Pépin L, Ritz C, Saltzman E, Stievenard M (1999). Climate and Atmospheric History of the Past 420,000 years from the Vostok Ice Core, Antarctica. Nature.

[ref-78] PRISM Climate Group (2004). Oregon State University. http://prism.oregonstate.edu.

[ref-110] R Core Team (2014). R: a language and environment for statistical computing.

[ref-79] Retallack GJ (2001). A 300-million-year record of atmospheric carbon dioxide from fossil plant cuticles. Nature.

[ref-81] Roth-Nebelsick A, Oehm C, Grein M, Utescher T, Kunzmann L, Friedrich JP, Konrad W (2014). Stomatal density and index data of *Platanus neptuni* leaf fossils and their evaluation as a CO_2_ proxy for the Oligocene. Review of Palaeobotany and Palynology.

[ref-82] Rubino M, Etheridge DM, Trudinger CM, Allison CE, Battle MO, Langenfelds RL, Jenk TM (2013). A revised 1000 year atmospheric δ^13^C-CO_2_ record from Law Dome and South Pole, Antarctica. Journal of Geophysical Research: Atmospheres.

[ref-83] Schmitt J, Schneider R, Elsig J, Leuenberger D, Lourantou A, Chappellaz J, Köhler P, Joos F, Stocker TF, Leuenberger M, Fischer H (2012). Carbon isotope constraints on the deglacial CO_2_ rise from ice cores. Science.

[ref-84] Schubert BA, Jahren AH (2012). The effects of atmospheric CO_2_ concentration on carbon isotope fractionation in C_3_ land plants. Geochimica et Cosmochimica Acta.

[ref-85] Schubert BA, Jahren AH (2018). Incorporating the effects of photorespiration into terrestrial paleoclimate reconstruction. Earth-Science Reviews.

[ref-91] Talhelm AF, Pregitzer K, Kubiske M, Zak D, Campany C, Burton A (2013). Elevated CO_2_ and O_3_ alter productivity and carbon storage in Northern Temperate Forests: results from Aspen FACE.

[ref-93] Tieszen LL (1991). Natural variations in the carbon isotope values of plants: implications for archaeology, ecology, and paleoecology. Journal of Archaeological Science.

[ref-94] Tipple BJ, Meyers SR, Pagani M (2010). Carbon isotope ratio of Cenozoic CO_2_: a comparative evaluation of available geochemical proxies. Paleoceanography.

[ref-95] Tognetti R, Minnocci A, Penuelas J, Rachi A, Jones MB (2000). Comparative field water relations of three Mediterranean shrub species co-occurring at a natural CO_2_ vent. Journal of Experimental Botany.

[ref-96] Troughton JH, Card KA (1975). Temperature effects on the carbon-isotope ratio of C_3_, C_4_ and Crassulacean-acid-metabolism (CAM) plants. Planta.

[ref-97] Trudinger CM, Enting IG, Francey RJ, Etheridge DM, Rayner PJ (1999). Long-term variability in the global carbon cycle inferred from a high-precision CO_2_ and δ^13^C ice-core record. Tellus B: Chemical and Physical Meteorology.

[ref-98] Van de Water PK, Leavitt SW, Betancourt JL (2002). Leaf δ^13^C variability with elevation, slope aspect, and precipitation in the southwest United States. Oecologia.

[ref-108] Wang R, Yu G, He N, Wang Q, Zhao N, Xu Z, Ge J (2015). Latitudinal variation of leaf stomatal traits from species to community level in forests: linkage with ecosystem productivity. Scientific Reports.

[ref-99] Wehr R, Saleska SR (2015). An improved isotopic method for partitioning net ecosystem-atmosphere CO_2_ exchange. Agricultural and Forest Meteorology.

[ref-100] Wernerehl RW, Givnish TJ (2015). Relative roles of soil moisture, nutrient supply, depth, and mechanical impedance in determining composition and structure of Wisconsin prairies. PLOS ONE.

[ref-101] White JWC, Vaughn BH, Michel SE (2015). University of Colorado, Institute of Arctic and Alpine Research (INSTAAR), stable isotopic composition of atmospheric carbon dioxide (^13^C and ^18^O) from the NOAA ESRL carbon cycle cooperative global air sampling network, 1990–2014.

[ref-102] Wickham H (2016). ggplot2: elegant graphics for data analysis.

[ref-103] Woodward FI (1987). Stomatal numbers are sensitive to increases in CO_2_ from pre-industrial levels. Nature.

[ref-104] Woodward FI, Bazzaz FA (1988). The responses of stomatal density to CO_2_ partial pressure. Journal of Experimental Botany.

[ref-105] Yonetani T, Gordon HB (2001). Simulated changes in the frequency of extremes and regional features of seasonal/annual temperature and precipitation when atmospheric CO_2_ is doubled. Journal of Climate.

[ref-107] Zhang YG, Pagani M, Liu Z, Bohaty SM, DeConto R (2013). A 40-million-year history of atmospheric CO_2_. Philosophical Transactions of the Royal Society: Mathematical, Physical and Engineering Sciences.

