# Supplementary figures and images for: Rapid response to anthropogenic climate change by Thuja occidentalis: implications for past climate reconstructions and future climate predictions
Source: PeerJ. 2019 Jul 26;7:e7378. doi: 10.7717/peerj.7378 (PMC6662565; doi:10.7717/peerj.7378)

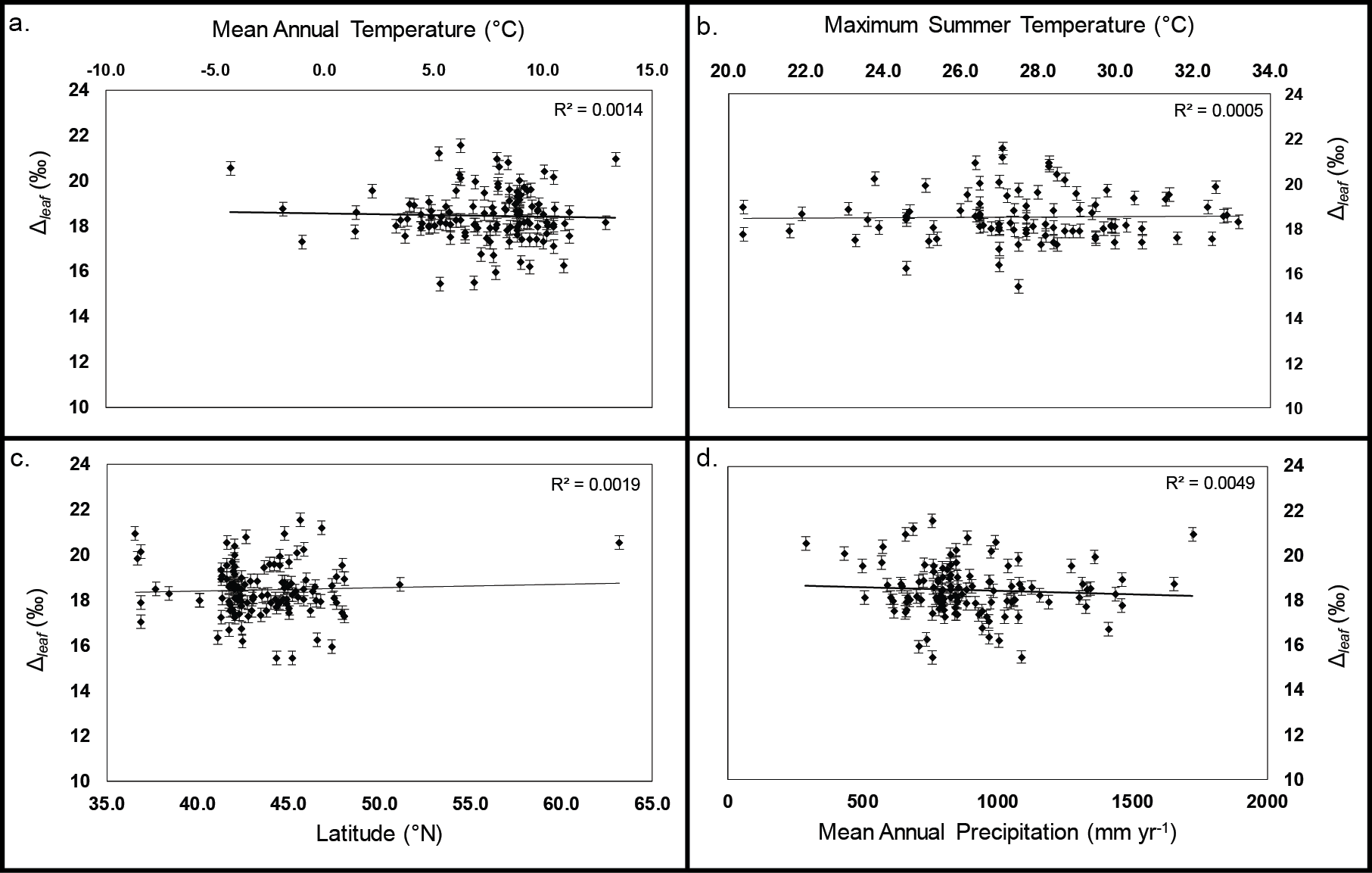

Supplement: Supplemental Information 1 — a) Δleaf values vs. mean annual temperature (−5 to 14°C) for collected specimens (R2 = 0.0001). b) Δleaf values vs. maximum summer (growing season) temperature (20 to 33°C) for collected specimens (R2 = 0.0005). c) Δleaf values vs. latitude (43 to 63°N) for collected specimens (R2 = 0.0002). d) Δleaf values vs. mean annual precipitation (288 to 1724 mm yr−1) for collected specimens (R2 = 0.0005). [file peerj-07-7378-s001.png]

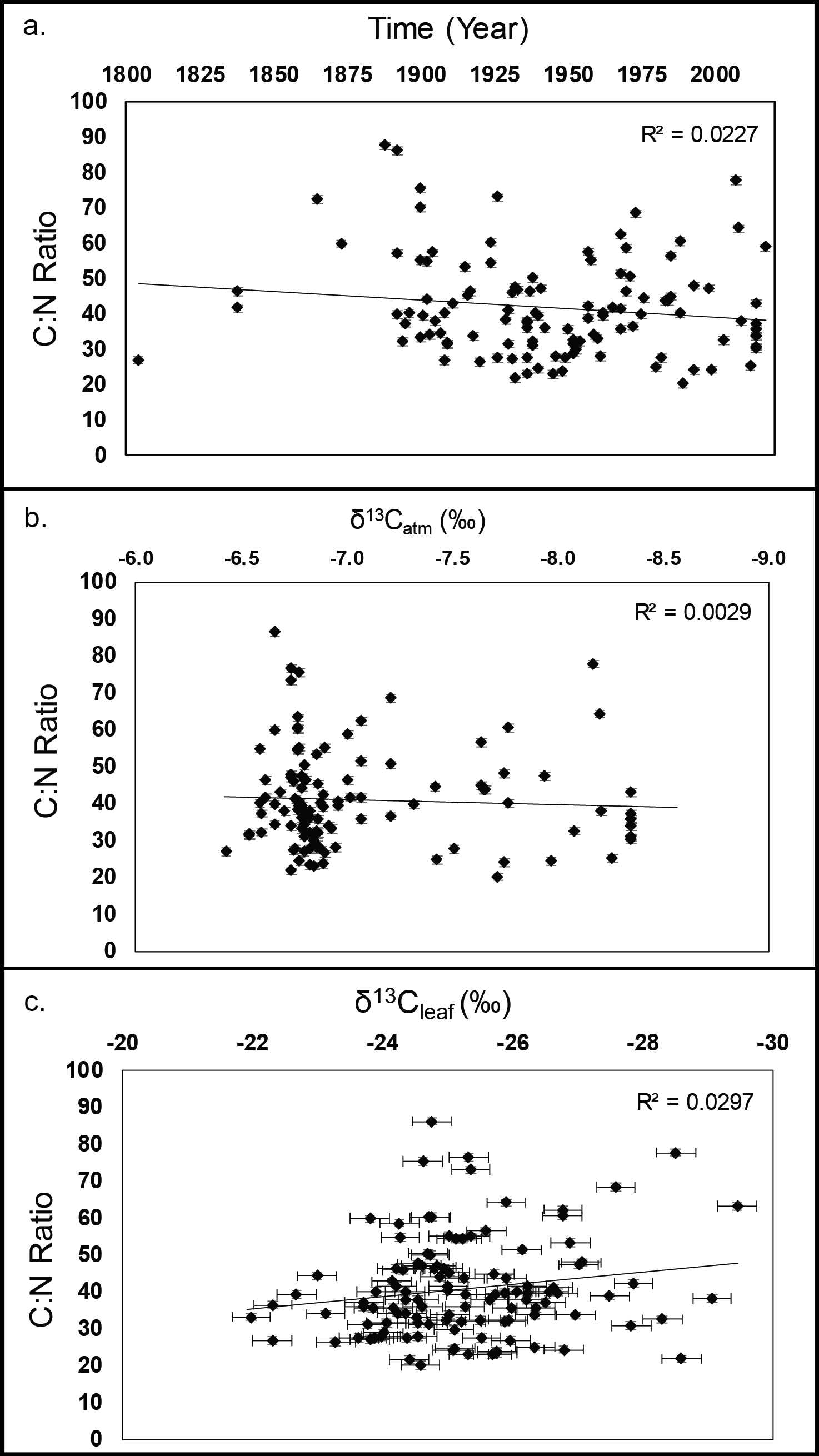

Supplement: Supplemental Information 2 — C:N ratios as determined by weight %C and weight %N measured on a Costech Elemental Analyzer compared to a) time collected (year), b) δ13Catm values (‰), c) δ13Cleaf values (‰). Error bars on the y-axis are associated with the 0.9% replicate reproducibility of standards. Error bars on the x-axis in c) are associated with the 0.3‰ replicate reproducibility of the Picarro CRDS standards. [file peerj-07-7378-s002.png]

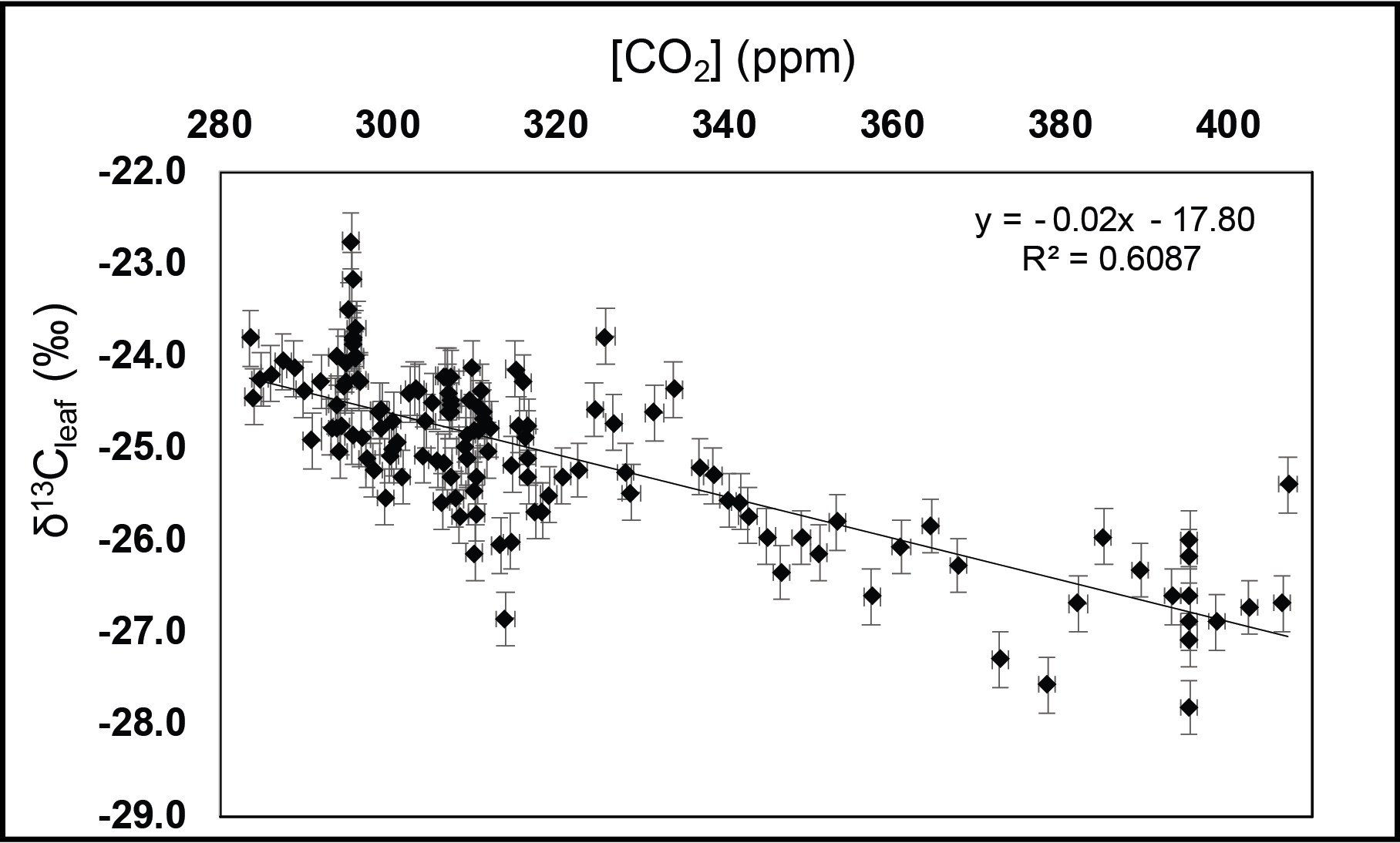

Supplement: Supplemental Information 3 — Linear regression between δ13Cleaf values (‰) and [CO2] (283 to 407 ppm) for collected specimens (R2 = 0.61). Error bars along the y-axis represent the ±0.3‰ replicate reproducibility of standards. [file peerj-07-7378-s003.png]

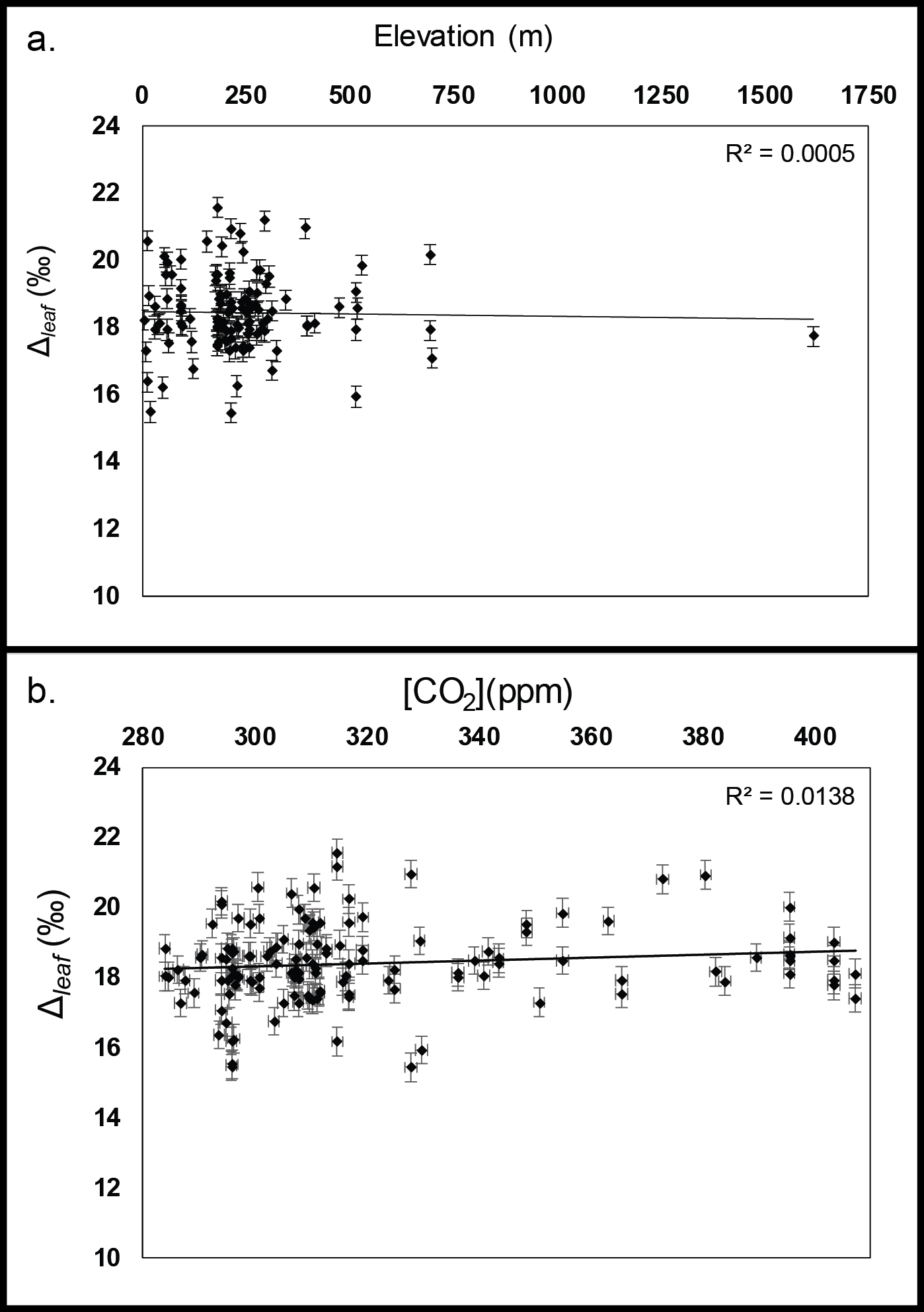

Supplement: Supplemental Information 4 — a) Linear regression between Δleaf values vs. elevation (4 to 1617 m above sea level). Error bars along the y-axis represent the ±0.3‰ replicate reproducibility of standards b) Δleaf and pCO2 (283 to 407 ppm) for collected specimens (R2 = 0.01). Error bars along the y-axis represent the ±0.3‰ replicate reproducibility of standards. [file peerj-07-7378-s004.png]

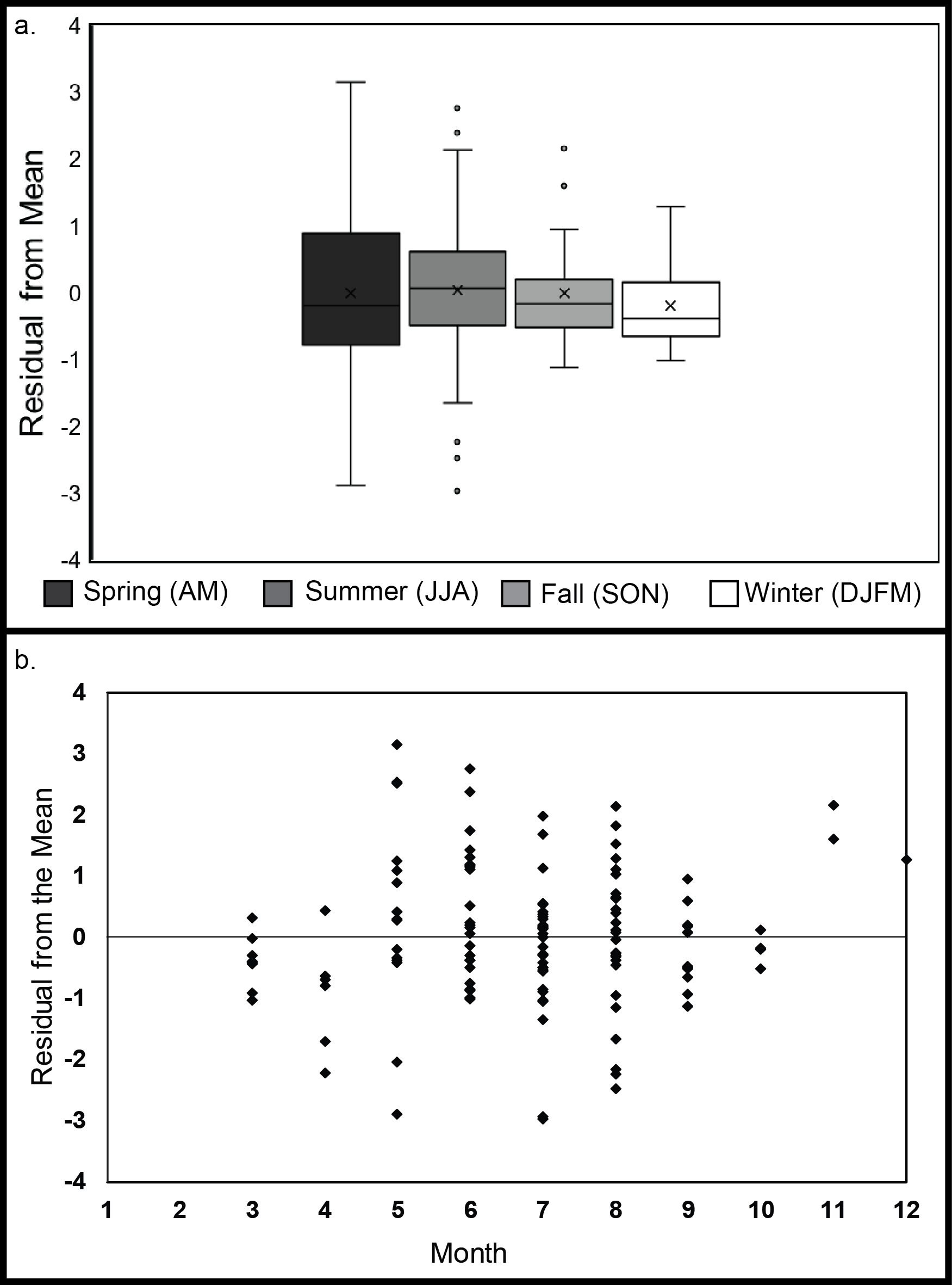

Supplement: Supplemental Information 5 — a) Box and Whisker plot showing median (lines), mean and range of spring, summer, fall and winter residual values of δ13Cleaf. T-tests assuming unequal variance comparing Spring vs. Summer, Fall, Winter, Summer vs. Fall, Winter, and Fall vs. Winter could not prove a null hypothesis; that the mean for all seasonal collections was significantly different. Seasons were categorized by mean monthly temperatures <°2C, from 2–15°C and rising, >15°C, and between 2–15°C and falling in the Great Lakes Region (centralized lower Peninsula Michigan), b) Scatter plot including residual from mean δ13Cleaf values as divided by month. [file peerj-07-7378-s005.png]

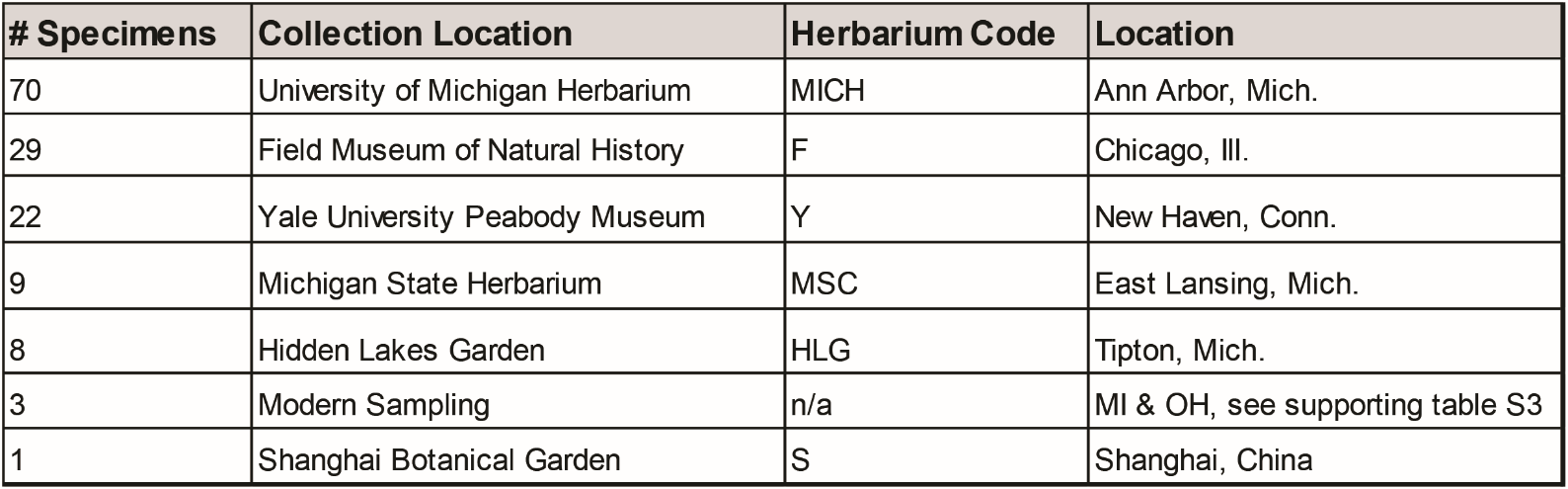

Supplement: Supplemental Information 6 [file peerj-07-7378-s006.docx]

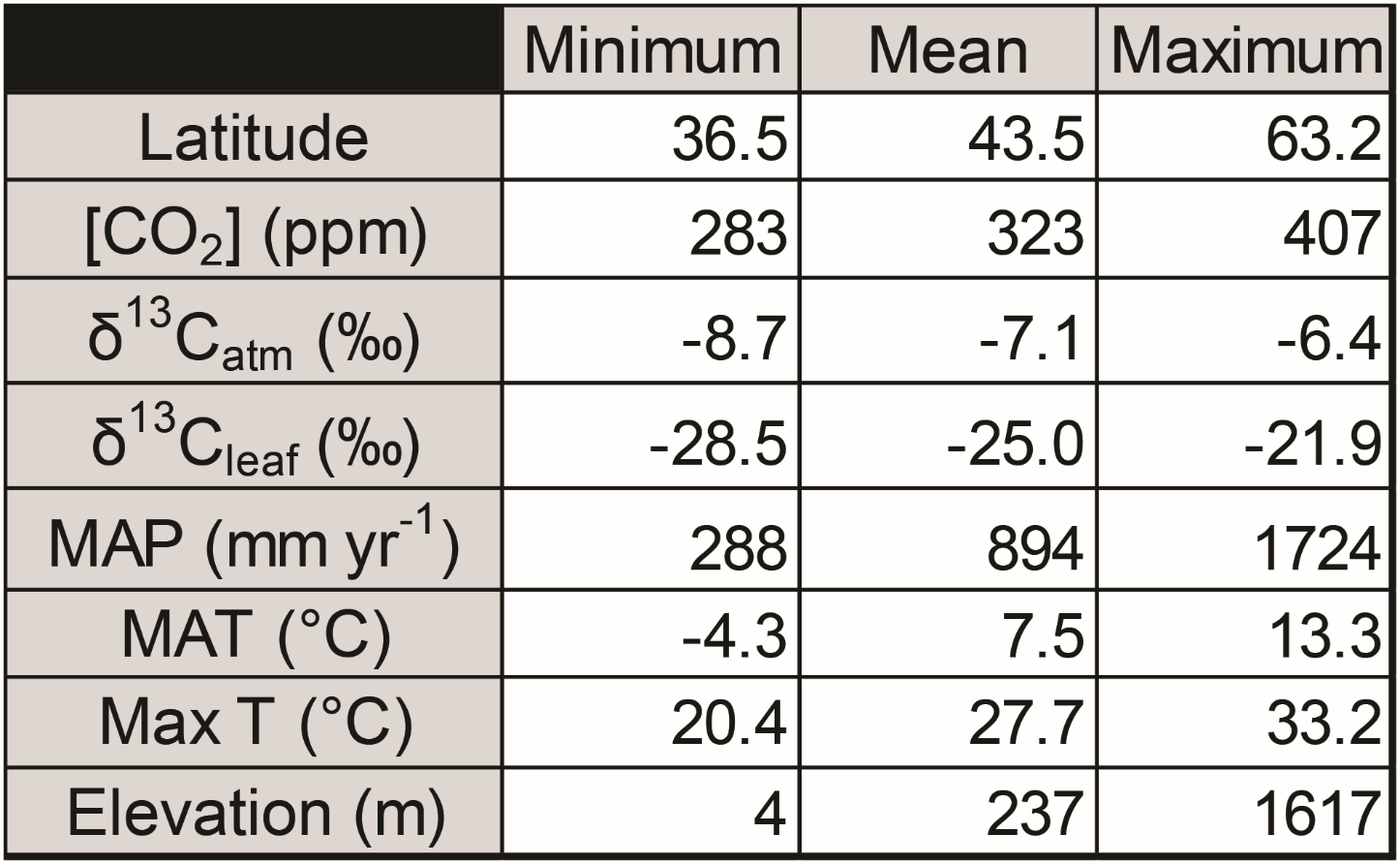

Supplement: Supplemental Information 7 — The minimum, maximum, and mean values for climate conditions for each specimen as obtained from NOAA ESRL Global Monitoring Division (2016), White et al. 2015, and PRISM Climate Group, as well as Government of Canada (Ed.). (2018, January 11). [file peerj-07-7378-s007.docx]
