# Supplementary material for: Rapid response to anthropogenic climate change by Thuja occidentalis: implications for past climate reconstructions and future climate predictions
Source: PeerJ. 2019 Jul 26;7:e7378. doi: 10.7717/peerj.7378 (PMC6662565; doi:10.7717/peerj.7378)
Supplement: Supplemental Information 9 [file peerj-07-7378-s009.docx]

world <- map_data("world")

usa <- map_data("usa")

points2 <- read.csv("latlongusa.csv", header=TRUE, sep=",")

points2

gg2 <- ggplot() + geom_polygon(data = world, aes(x=long, y =lat, group = group), fill = "white", color = "black") +

coord_fixed(xlim = c(-100, -65), ylim = c(25, 50), ratio = 1.2)

gg2 +

geom_point(data = points2, aes(x = longitude, y = latitude), color = "black", size = 2) +

geom_point(data = points2, aes(x = longitude, y = latitude), color = "chartreuse4", size = 1)
